# Rational Design of Non-Toxic Multidrug Combinations Demonstrates Durable and Hypoxia-Enhanced Efficacy Against Renal Cell Carcinoma

**DOI:** 10.3390/pharmaceutics17101269

**Published:** 2025-09-27

**Authors:** Valentin Mieville, Jakub Gubala, Mathis Fiault, Marie Ota, Seungsu Han, Muriel Urwyler, Daniel Benamran, Jean-Christophe Tille, Massimo Valerio, Patrycja Nowak-Sliwinska

**Affiliations:** 1 School of Pharmaceutical Sciences, Faculty of Science, University of Geneva, 1211 Geneva, Switzerland; valentin.mieville@unige.ch (V.M.); jakub.gubala@unige.ch (J.G.); mathis.fiault@unige.ch (M.F.); marie.ota@etu.unige.ch (M.O.); hansuziehan@gmail.com (S.H.); muriel.urwyler@unige.ch (M.U.); 2 Institute of Pharmaceutical Sciences of Western Switzerland, University of Geneva, 1211 Geneva, Switzerland; 3 Translational Research Center in Oncohaematology, 1211 Geneva, Switzerland; 4 Division of Urology, Geneva University Hospitals, 1211 Geneva, Switzerland; daniel.benamran@hug.ch (D.B.); massimo.valerio@hug.ch (M.V.); 5 Division of Pathology, Geneva University Hospitals, 1205 Geneva, Switzerland; jean-christophe.tille@hug.ch

**Keywords:** ccRCC, chRCC, drug combinations, hypoxia, patient-derived organoids, RCC, safety, screening, spheroids

## Abstract

**Background/Objectives**: Despite recent therapeutic advances, the clinical management of renal cell carcinoma (RCC) remains suboptimal. Current treatments are hindered by limited efficacy, the emergence of acquired drug resistance, suboptimal tolerability, and a lack of tumor-specific targeting. While development of novel agents remains an important avenue, it is often constrained by high costs, long development time, and low success rates. As an alternative approach, drug combinations of approved agents offer a promising strategy. **Methods**: Using our proprietary drug combination methodology, we identified multidrug combinations in RCC cells representing the clear cell (786O) and sarcomatoid chromophobe (UOK276) histological subtypes of RCC. **Results**: From an initial panel of 10 drugs, either approved or undergoing clinical trial, the optimized drug combinations (ODCs) contained crizotinib, telaglenastat, U-104, and vismodegib at clinical and subtherapeutic doses. The ODCs were non-toxic in advanced hepatic, renal, and cardiac cellular models. Importantly, their anti-tumor activity, already notable in normoxic (21% O_2_) conditions (approx. 50%) was markedly enhanced in tumor-relevant hypoxia (1.5% O_2_), reaching up to 77% in 2D and 62% in 3D spheroid 786O models. Moreover, chronic exposure of 786O and UOK276 cells led to durable responses, suggesting a prolonged effect in responders. **Conclusions**: Our findings demonstrate the potential of optimized, non-toxic drug combinations as a highly selective and effective strategy for accelerating the development of precision RCC treatment.

## 1. Background

Despite the development of targeted and immune-based therapies, the clinical management of many cancers, including renal cell carcinoma (RCC), remains challenging [1,2]. Although current therapeutic options have improved overall patient survival, they are frequently associated with significant toxicity and limited durability of the response [2,3]. Furthermore, a significant fraction of patients exhibits either innate or acquired resistance to first-line therapies, necessitating transition to subsequent treatment lines [2,4]. However, for RCC, these second-line treatment options remain limited to drugs with similar mechanisms of action, primarily tyrosine kinase inhibitors (TKIs) targeting vascular endothelial growth factor receptors (VEGFR) and immune checkpoint inhibitors [5]. Although many patients show initial responsiveness to second-line therapy, the majority ultimately experience disease progression, further limiting treatment options [5,6,7]. This highlights a serious need for new therapeutic alternatives effective against RCC. Drug development is a critical but lengthy and costly process that frequently fails to give satisfactory results [8,9].

In parallel, an alternative and complementary strategy of combining existing drugs has emerged [10,11]. Numerous studies have demonstrated that drugs with suboptimal activity as single agents can achieve important anti-neoplastic effects when combined [11,12,13,14,15]. This strategy offers a clinically and economically interesting alternative to de novo drug development by leveraging approved medications [10]. However, the identification of optimal drug combinations presents substantial challenges. Firstly, as for single agents, patient-specific factors can influence response to a combination [16,17,18]. Secondly, drugs may have positive (synergistic), neutral (additive), but also detrimental (antagonistic) interactions, requiring careful selection and interaction analysis [16,17,18,19]. Moreover, it should be noted that while interactions can significantly improve antitumor efficacy, they can also induce substantial off-target toxicity [19]. Finally, the large number of marketed drugs, especially when combining repurposed agents with existing anticancer drugs, create an almost limitless array of combinatorial possibilities [20].

Our research group developed a proprietary phenotypically guided approach to rapidly identify multidrug combinations from a defined drug set. This approach, named Therapeutically Guided Multidrug Optimization (TGMO), has already proven its effectiveness in finding effective drug combinations in multiple cancer types [12,13,14,15,21,22]. TGMO is a statistics-driven framework designed to enhance precision medicine by systematically optimizing multidrug combinations in laboratory settings. It integrates statistics to predict synergistic drug interactions, computational modelling to balance efficacy and toxicity, and patient-specific biomarker profiling for personalized therapy design. TGMO dynamically adjusts treatment regimens based on real-time, clinically valid response, ensuring adaptive precision. Following this methodology, drugs negatively contributing to the safety and efficacy of combinations are weeded off and only safe drugs presenting antitumoral synergistic and additive interactions remain in the final combination. This approach maximizes therapeutic outcomes while minimizing adverse effects, offering a robust solution for next-generation combinatorial treatment strategies. While mechanistic knowledge of the drugs (e.g., targets, pathways) can enhance TGMO by improving interpretability and biological plausibility, it is not strictly required. TGMO can infer optimal drug combinations directly from high-throughput in vitro data or clinical outcomes, even without prior mechanistic information. The ultimate result of the optimization depends on the initial drug selection and their combined effects on the phenotype of the selected biological models.

Our initial drug set was strategically selected to target a diverse array of core hallmarks and enabling characteristics of cancer involved in RCC progression and therapy resistance. The selection was based on two principles: (i) including drugs with documented preclinical or clinical activity in RCC, and (ii) ensuring a wide coverage of non-overlapping, complementary mechanisms of action to favour the potential for synergistic suppression of tumor growth and viability. Axitinib was included as a benchmark first-line RCC treatment targeting angiogenesis primarily through VEGFR inhibition, with additional activity against PDGFR and c-KIT relevant for tumor stroma interactions [5,23]. Osimertinib (EGFR inhibitor) and selumetinib (MEK inhibitor) were selected to inhibit the MAPK pathway, a key signaling cascade frequently dysregulated in RCC. Their high selection rate in our previous TGMO screens on RCC models refs provided a strong empirical basis for their inclusion [11,13].

Crizotinib was chosen for its potent inhibition of c-Met, a receptor tyrosine kinase often overexpressed or activated in RCC and associated with aggressive disease and resistance to anti-VEGF therapies [24]. Telaglenastat (CB-839), a glutaminase inhibitor, was included to exploit the well-documented glutamine addiction of many RCC tumors, disrupting their energy production and biosynthetic capabilities [25]. U-104, a potent inhibitor of carbonic anhydrase IX (CAIX), targets a key enzyme induced by tumor hypoxia. CAIX is crucial for maintaining intracellular pH and promoting survival in the acidic tumor microenvironment, a hallmark of RCC [26,27]. Palbociclib (CDK4/6 inhibitor) was selected to directly induce cell cycle arrest, targeting the dysregulated cyclin D-CDK4/6-Rb pathway observed in various cancers [27,28]. Vismodegib (Hedgehog pathway inhibitor) was included to target a developmental pathway implicated in cancer stem cell maintenance, tumor-stroma crosstalk, and involved in RCC tumors [29,30,31]. Finally, we selected drugs targeting inflammation (aspirin) and cholesterol homeostasis (simvastatin). Aspirin, a COX inhibitor, was chosen for its anti-inflammatory properties, targeting the pro-tumorigenic microenvironment, and for its emerging epidemiological links to reduced cancer risk [27,32]. Simvastatin (HMG-CoA reductase inhibitor) was included to disrupt cholesterol biosynthesis, which is critical for cell membrane integrity and signaling pathways, and has demonstrated anti-proliferative effects in RCC models [33]. The TGMO platform was then used to identify the most synergistic and safe drug combinations.

In the present study, we performed the TGMO-based screen using a clear cell RCC (ccRCC) cell line (786O), and a chromophobe RCC (chRCC) cell line (UOK276). 786O is a human cell line originating from a primary ccRCC tumor. It is a well-studied aggressive cell line characterized by mutations affecting VHL, PTEN, and TP53—all relatively common in ccRCC tumors [34,35]. UOK276 is one of the only characterized chRCC cell lines. UOK276 is derived from a sarcomatoid region of a primary chRCC tumor, a differentiation that is linked to a particularly aggressive phenotype. Mutations in UOK276 cells were identified in TP53, TRAF7, SMARCA4, and SMO genes [30].

According to European guidelines, the treatment strategy for both cell lines would be similar, as algorithms for sarcomatoid RCC and ccRCC contain the same treatment options [5]. However, these similar guidelines appear to be more influenced by the scarcity of chRCC/sarcomatoid RCC patients than by the actual efficacy of the therapy as implied by American recommendations [36]. Consequently, sarcomatoid RCC patients face a 5-year survival of only 18% in the US compared to an overall 74% for all RCC patients [37]. This highlights the critical need for new agents or drug combinations targeting this subtype of RCC.

Additionally, the ODCs were screened in non-cancerous renal epithelial cells (RPTEC). These proximal tubule cells act as our healthy control to detect and remove potentially toxic interactions. Safety profile was more broadly confirmed using cellular models of kidney (patient-derived organoids), liver (HepaRG), and heart (H9c2). In a previous study, these models demonstrated sensitivity to drug combination-related toxicity and known nephrotoxic, hepatotoxic, and cardiotoxic drugs [38].

Starting with our set of ten drugs, we attempted to find positively interacting (optimized) drug combinations (ODCs) effective on 786O, and UOK276 cell lines, but inactive on non-malignant cellular models. Using three experimental iterations and data modeling within TGMO, we identified three novel drug combinations selective toward cancer cells and inactive in RPTEC cells, and in cellular models of kidney, liver, and heart. ODCs gave a more pronounced effect in tumor-relevant hypoxic and three-dimensional conditions. A preliminary investigation of the mechanism of action demonstrated influence on the cell cycle distribution and cell death induction in 786O cells. While limited activity was highlighted in some patient-derived models, suggesting patient-specific benefits, the tumor specificity and durable effects demonstrated by those ODCs could make them appealing and safe options against renal cell carcinoma management.

## 2. Methods

### 2.1. Cell Culture

786O cells (ATCC, CRL-1932) were cultivated in RPMI medium with Glutamax (ThermoFisher Scientific, Waltham, MA, USA, 61870-010) supplemented with 10% fetal bovine serum (FBS; Biowest, Nuaillé, France, S1810-500) and 1% penicillin/streptomycin (Sigma-Aldrich, Saint-Louis, MO, USA, P0781-100ML). UOK276 cells were courtesy of W. Marston Linehan (National Cancer Institute, Bethesda, MD, USA) and were cultivated in Dulbecco’s modified eagle medium (DMEM) with high glucose and L-Glutamine but without Na Pyruvate (ThermoFisher Scientific, Waltham, MA, USA, 41965-039), supplemented with 10% FBS and 1% penicillin/streptomycin. RPTEC (TERT1) cells (ATCC, CRL-4031) were cultivated in complete Renal Epithelial Cell Growth medium 2 (PromoCell^®^, C-26030) supplemented with 1% penicillin/streptomycin. HepaRG cells (Biopredic International, Saint-Grégoire, France, HPR101) were acquired with the kind agreement of Dr. Guguen-Guillouzo, Dr. Gripon, and Dr. Trepo [39] and cultured in William’s E medium (BioConcept Ltd., Allschwil, Switzerland, 1-48F02-I) supplemented with 10% FBS, 1% L-glutamine (ThermoFisher Scientific, Waltham, MA, USA, 25030024), 1% penicillin/streptomycin, 50 μM hydrocortisone 21-hemisuccinate (Cayman Chemical Company, Ann Arbor, MI, USA, 18226), and 5 μL/mL insulin (Sigma-Aldrich, Saint-Louis, MO, USA, I9278-5ML). H9c2 cells (ATCC, Manassas, VA, USA, CRL-1446) were kindly gifted by Prof. Brenda Kwak from the Department of Pathology and Immunology at the University of Geneva, and cultured in DMEM (ThermoFisher Scientific, Waltham, MA, USA, 31966-021) supplemented with 10% FBS and 1% penicillin/streptomycin. Culture and differentiation processes are detailed in a previous publication [38]. Patient-derived kidney materials were isolated and handled as detailed in the published protocol [40]. All cells were maintained at 37 °C and 5% CO_2_.

Patient-derived cancer cells (PRCC1, PRCC9, PRCC18, and PRCC20) were isolated in 3D (PRCC1 and PRCC9) or in 2D (PRCC18 and PRCC20) following our previously published protocol [40]. PRCC17_1 were generously donated by our collaborators from the Department of Pathology and Molecular Pathology at the Universität’s Spital Zürich [41]. PRCC1, PRCC9, and PRCC17_1 were maintained and expanded into Matrigel^®^ (Corning Life Sciences B.V., Amsterdam, The Netherlands) domes with Renal Epithelial Cell Growth Medium 2 (PromoCell^®^, C-26030). PRCC18 and PRCC20 cells were maintained in tissue culture flasks (TPP Techno Plastic Products AG, Trasadingen, Switzerland, 90026 and 90076) with the Renal Epithelial Cell Growth Medium 2. A detailed description of all procedures can be found in a previously published article [40].

### 2.2. Seeding Procedure

For two-dimensional assays in flat-bottom 96-well plates (Greiner Bio-One, Kremsmünster, Austria, 655180), cells were plated 24 h before treatment at a density of 2500 (786O), 3000 (RPTEC), or 4000 (UOK276) cells/well. For assays in T25 tissue culture flasks (TPP Techno Plastic Products AG, Trasadingen, Switzerland, 90026), cells were seeded at densities of 200,000 (786O) or 312,500 (UOK276) cells/flask.

For three-dimensional assays, cells were seeded 96 h before treatment at a density of 1000 cells/well in U-bottom ultra-low attachment 96-well plates (Greiner Bio-One, Kremsmünster, Austria, 650970). For this procedure, cells were suspended in culture medium containing 2.5% of Matrigel^®^ (Corning Life Sciences B.V., Amsterdam, The Netherlands) and were centrifuged in the plates for 5 min at 400× *g*, 4 °C.

### 2.3. Drugs

Aspirin (HY-14654), axitinib (HY-10065), bortezomib (HY-10227), crizotinib (HY-50878A), osimertinib (HY-1772A), palbociclib (HY-50767), selumetinib (HY-50706), simvastatin (HY-17502), telaglenastat (HY-12248), U-104 (HY-13513), vismodegib (HY-10440), cisplatin (HY-17394), and doxorubicin (HY-15142) were all purchased from MedChemTronica (Sollentuna, Sweden). Cisplatin was dissolved in DMF (ThermoFisher Scientific, Waltham, MA, USA, 21058) and conserved at −80 °C. All other drugs were dissolved in anhydrous DMSO (Sigma-Aldrich, Saint-Louis, MO, USA, 276855) and conserved at −80 °C. All drugs were conserved and aliquoted in single-use volumes for a maximum duration of 6 months. Cisplatin, simvastatin, and doxorubicin were used as positive controls for nephrotoxicity, hepatotoxicity, and cardiotoxicity, respectively [38].

### 2.4. Treatment in Hypoxic Conditions

Plated cells and spheroids were transferred into an InvivO2 400 hypoxia chamber (The Baker Company Inc., Sanford, ME, USA) set at 1.5% O_2_, 5% CO_2_, and 37 °C. Drug mixtures were prepared in atmospheric conditions and then equilibrated to the hypoxic environment for 30 min before addition to the cells. Treated plates were retrieved from the hypoxia chamber immediately before performing the cell viability assay.

### 2.5. Cell Viability Assays

The activity of drug treatments on 2D and 3D cell culture models was determined using 2D (G7572), and 3D (G9683) CellTiter-Glo^®^ Luminescent Cell Viability assays (CTG; Promega, Dübendorf, Switzerland), respectively. CTG assay gives indirect cell viability information through the semi-quantitative bioluminescent measurement of cellular ATP. Reagents was added directly to each well of the 96-well plates. Plates were then incubated in the dark at room temperature for complete cell lysis. At the end of the 30 min incubation, the cell lysates were homogenized and transferred into flat, clear-bottom, black side 96-well plates (Falcon, 353219). Bioluminescence readout was performed using a Cytation 5 plate reader (BioTek Instruments, Winooski, VT, USA) and Gen 5 software (v. 3.15) at default luminescence, endpoint kinetic settings.

Cell counting with Trypan blue exclusion dye was performed as an orthogonal viability assay and as the primary readout method of the repeated exposure experiments. Cells were harvested using 0.05% trypsin-EDTA with phenol red (ThermoFisher Scientific, Waltham, MA, USA, 25300054). Cell suspensions were diluted 1:1 in Trypan blue solution 0.4% (ThermoFisher Scientific, Waltham, MA, USA, 1520061) before numeration with a Neubauer counting chamber. Both CTG and cell counting assays were performed in accordance with the instructions of Promega and ThermoFisher Scientific, providing respectively the CTG and Trypan blue reagents.

### 2.6. TGMO Methodology

This approach, patented by Patrycja Nowak-Sliwinska and colleagues (Patent No: US20220373535A1 and (Weiss et al. 2019) [13]), is designed to overcome the fundamental challenge of searching the exponentially large parameter space of multidrug combinations. This process allows for the highly efficient identification of optimal combinations with far fewer experiments than a full factorial screening. The TGMO is divided into the following steps performed iteratively (Supplementary Figure S1): An initial screen of individual compounds in cancerous and non-cancerous cells to identify low (IC_20_) doses and large therapeutic windows; A fraction of the possible drug combination doses is tested in a relevant biological assay (e.g., cell metabolic activity). This is done based on the design of the experiment, which defines a large set of drug combinations tested experimentally (Supplementary Figures S3–S8). The experimental results (cell metabolic activity as a % of CTRL) obtained in cancerous and non-cancerous cells are used to build a predictive model of the entire dose–response surface. This model (Matlab^®^, v. R2023b) using second-order step-wise linear regression models is then used to guide the selection of the next, most informative set of combinations to test, focusing the search on regions of the dose-space that align with pre-defined therapeutic goals (e.g., high synergy, low cytotoxicity on non-cancerous cells). The reliability of regression models was further confirmed based on analysis, including the elimination of outlier data points (Cook’s distance), the root mean square error, and the evaluation of the coefficient of multiple determination, as well as the correlations between fitted and observed data points (Supplementary Figure S9). The selection of an appropriate model structure was confirmed with an ANOVA test. Regression models were used to eliminate compounds from the search, leading to the subsequent search. The drugs demonstrating antagonism with other tested medications are eliminated (Supplementary Figures S3–S8). The next set of experiments is conducted using the same principle but with the narrowed set of drugs (Supplementary Figures S10–S12). The data are modelled again, and the only 4 drugs presenting synergistic or additive drug–drug interactions and lack of toxicity are enrolled in the last optimization round, where the drug optimal doses are further confirmed.

### 2.7. Re-Treatment Experiments

In the first experiment, the cells were seeded in 2D in 2 different 96-well plates and treated after 24 h. After a first 72-h exposure to the different drugs, one plate was read out using CTG. In the other plate, the liquid in each well was replaced with freshly prepared treatment conditions. The second treatment was left to act for another 72 h, then the plate was read out using CTG.

In the second experiment, cells were seeded in T25 flasks and treated after 24 h. After a 72-h exposure time, the cells were harvested from each T25 and seeded in one 96-well plate per pre-treated condition. After 24 h, the plates were treated with all different treatment conditions, and the readout was performed 72 h later using CTG.

For the multiple retreatment experiment, cells were seeded and treated in T25 culture flasks as mentioned above. After initial retreatment, cells were seeded in new T25s every Monday, treated every Tuesday, and counted every Friday. From Fridays to Mondays, the cells were seeded in T25s without treatment. Readout for this experiment was initiated with manual counting using, at first, the Neubauer counting chamber and then a Countess automatic cell counter (ThermoFisher Scientific, Waltham, MA, USA), as the latter demonstrated similarly robust results obtained more rapidly.

The various re-treatment procedures were schematically illustrated in Section 3.4.

### 2.8. Cell Cycle Distribution Analysis

786O and UOK276 cells were exposed to treatments in T25 tissue culture flasks (TPP Techno Plastic Products AG, Trasadingen, Switzerland, 90026) for 24 h. After treatment, cells were harvested using 0.05% trypsin-EDTA with phenol red (ThermoFisher Scientific, Waltham, MA, USA, 25300054), washed twice with phosphate-buffered saline, pH 7.4, without calcium, magnesium or phenol red (PBS; ThermoFisher Scientific, Waltham, MA, USA, 10010015), then fixed in 70% ethanol solution (diluted from Sigma-Aldrich, Saint-Louis, MO, USA, 51976-500ML) at 4 °C. Fixed cells were kept at 4 °C until use [42]. Propidium iodide (PI) staining was performed 24 h before flow cytometry readout. Cells were washed twice with PBS, then suspended in FxCycle PI/RNase staining solution (ThermoFisher Scientific, Waltham, MA, USA, F10797) following the instructions of the provider. Cells in the staining solution were kept in the dark at 4 °C until the readout. Analysis was performed on a CytoFLEX flow cytometer (Beckman Coulter, Pasadena, CA, USA). Cell cycle distribution analysis was completed using FlowJo Single Cell Analysis Software (v. 10). An example of the gating strategy can be found in Supplementary Figure S17.

### 2.9. Cell Death Assay

786O and UOK276 cells were exposed to treatments in T25 tissue culture flasks (TPP Techno Plastic Products AG, Trasadingen, Switzerland, 90026) for 24 h. Bortezomib at 10 μM was used as a positive control for the assay. After treatment, the medium was collected to ensure all dead cells were considered. Then, cells were harvested using 0.05% trypsin-EDTA with phenol red (ThermoFisher Scientific, Waltham, MA, USA, 25300054), and the collected medium was transferred to an adequate cell suspension. Cells were then centrifuged at 300× *g* for 5 min, washed twice with cold phosphate-buffered saline, pH 7.4, without calcium, magnesium or phenol red (PBS; ThermoFisher Scientific, Waltham, MA, USA, 10010015), and resuspended in 1 mL of PBS. Cells were counted and 200,000 cells each were transferred into 5 mL Polystyrene Round-Bottom Tubes (Corning Life Sciences B.V., Amsterdam, The Netherlands, 352058). Cells were then stained with BD Pharmingen™ FITC Annexin V Apoptosis Detection Kit I (BD Biosciences, San Diego, CA, USA, 556547) following the provider’s instructions. Cells in the staining solution were kept in the dark at 4 °C until the readout. Analysis was performed on a CytoFLEX flow cytometer (Beckman Coulter, Pasadena, CA, USA). Cell cycle distribution analysis was completed using FlowJo Single Cell Analysis Software (v. 10). An example of the gating strategy can be found in Supplementary Figure S16C,D.

### 2.10. RNA Sequencing

Cells were treated in T25 flasks (TPP Techno Plastic Products AG, Trasadingen, Switzerland, 90026) for 72 h before being harvested using 0.05% trypsin-EDTA with phenol red (ThermoFisher Scientific, Waltham, MA, USA, 25300054), and then washed once with PBS (ThermoFisher Scientific, Waltham, MA, USA, 10010015). RNA was isolated using the RNeasy^®^ Micro kit (Qiagen, Hilden, Germany, 74104) according to the manufacturer’s instructions. Analysis was performed by the Genomic platform at the University of Geneva. RNA was sequenced on an Illumina NovaSeq 6000 using Illumina Stranded mRNA Prep, 100 single-end reads protocol. Quality control was performed with FastQC (v. 0.11.9). All reads were mapped to reference human genome GRCh38 using STAR RNA-seq aligner (v. 2.7.10b) [43]. Biological quality control and summarization were carried out with PicardTools (v. 2.21.6). Raw counts were acquired using HTseq v. 0.11.3. The R package edgeR 3.42.4 was used to perform normalization and differential expression analysis of the annotated genes [44]. Significance between experimental factors was assessed using a general linear model, a quasi-likelihood F-test, and a Benjamini & Hochberg test correction for false discovery rate. Genes were considered significantly different if the fold-change was greater than 2 and the corrected *p*-value was less than 0.05. Enrichment analysis was performed using the Enrichr web-based tool [45,46,47].

### 2.11. Statistical Analysis

Significance was calculated from at least three (N ≥ 3) independent experiments and was always calculated between indicated conditions using the GraphPad Prism^®^ software (v. 10.4.1). Statistical tests used are indicated below each figure. Error bars correspond to the standard deviations, and the *p*-value is specified in each figure as ^ns^ *p* > 0.05, * *p* < 0.5, ** *p* < 0.01, *** *p* < 0.001, or **** *p* < 0.0001.

## 3. Results

### 3.1. The TGMO-Based Optimization Identifies Novel Drug Combinations

In the first optimization step, each drug was tested at a range of concentrations in 786O and UOK276 cells. Dose–response curves were drawn based on cellular activity of the treated cells compared to the 0.1% DMSO vehicle control (Supplementary Figure S1A). Equations of the dose–response curves were calculated in GraphPad Prism^®^ using the embedded *Dose–response-inhibition* log(inhibitor) vs. normalized *response—Variable slope* analysis model. These equations were used to determine the concentration of each drug needed for a 20% inhibition of the ATP levels (Supplementary Figure S1B). The IC_20_ of each drug was selected for the combination screen. In cases when a dose–response curve was flat or if the calculated IC_20_ was above the clinically used dose (CUD; see Table 1), the CUD was used to perform the screening. CUDs were calculated using plasma AUC values from the literature or official documentation.

Using the TGMO-based matrices, originating from an orthogonal array composite design, several multidrug combinations were tested, both in RCC cells as well as in non-malignant RPTEC cells. During the initial experimental screen (search 1), osimertinib demonstrated significant toxicity (Supplementary Figure S2). Consequently, search 1 was repeated with reduced doses of osimertinib (from 0.469 μM to 0.059 μM). In this search, the metabolic activity in cancerous and non-malignant cells was reduced to a minimum of 51.1% in 786O cells (Supplementary Figure S3A), 60.0% in UOK276 cells (Supplementary Figure S6A), and 65.1% in RPTEC cells (Supplementary Figure S4A).

To further refine the drug pool, the effects of each drug and drug–drug interaction in our results were deconvoluted using TGMO-based data modeling. The effect observed in RPTEC cells was weighed against the effect observed on the cancer cells by subtracting the effect on the cancer cells from the effect on the healthy cells in each condition to obtain what is called here the therapeutic window (TW) of the drug combination. This TW and the effect measured on the cancer cell were entered separately into the TGMO-based MATLAB (v. 2023b) script to obtain the regression coefficients (Supplementary Figures S5A and S8A). A positive regression coefficient represents reduced activity in the cells. This is desirable for the TW but negative for the cancer cells. Oppositely, a negative regression coefficient means increased activity in the cells and is therefore sought in the cancer cells but undesirable on the TW. Following this logic, three drugs were removed based mainly on their drug–drug interactions but also on their distinct effect. In the 786O-based screen (Supplementary Figure S5A), aspirin, axitinib, and osimertinib displayed multiple negative drug–drug interactions and negatively impacted the anti-cancerous effect (aspirin and axitinib), or poor toxicity profiles alone (all three). In the UOK276-based screen (Supplementary Figure S8A), the three drugs removed were aspirin, osimertinib, and simvastatin. Even at a reduced dose, osimertinib exhibited only negative interaction and toxicity, while aspirin and simvastatin had little to no effect alone, while contributing mostly to negative drug–drug interactions.

After removal of the drugs, a new search (search 2) of the screen was performed following the same design with an adapted treatment matrix. Metabolic activity data of the 786O-based screen exhibited a decreased toxicity profile with a minimum of 78.1% between all combinations in RPTEC (Supplementary Figure S4B), but also a decrease in anticancer activity with a minimum of 66.1% (Supplementary Figure S3B). For the UOK276 screen, there was a net increase in the anticancer activity with a minimum of 47.2% (Supplementary Figure S6B), and a net decrease in toxicity with a minimum of 75.9% in RPTEC cells (Supplementary Figure S7A). Regression coefficient results (Supplementary Figures S5B and S8B) were used to further remove the drugs causing unfavorable drug–drug interactions. Axitinib, palbociclib, and selumetinib were removed from the UOK276-based screen. Simvastatin, U104, and vismodegib were removed from the 786O-based screen.

To assess whether the combination could benefit from drug concentration adaptation, a final experimental iteration (search 3) focused on dose optimization was performed. This search consisted of four drugs and was performed twice in parallel, i.e., (i) maintaining previous concentrations and (ii) including higher doses of the drugs, unless already used at CUD (Table 1). In iteration (ii), the dose of telaglenastat was increased to 0.013 µM, the dose of palbociclib was increased to 0.264 µM, the dose of U104 was increased to 9.524 µM, and the dose of vismodegib was increased to 12.754 µM. Starting with the 786O screen with the previously used dose (i) (Supplementary Figures S3C and S4C), the combination of the four drugs together decreased the metabolic activity of 786O cells to 73.2% of the control, while it reduced the metabolic activity of RPTEC cells to 79.1% of the control. The increased dose in the parallel 786O screen (ii) was beneficial for the TW as the effect of the increase was greater in cancer cells, with the four-drug combination decreasing the metabolic activity of 786O cells to 63.7% to the RPTEC cells, which only had a decrease to 77.2% (Supplementary Figures S3D and S4D). In the screen at the higher dose, we noticed that the interaction profile of palbociclib and selumetinib became detrimental to the combination (Supplementary Figure S5C,D).

**Figure 1:**
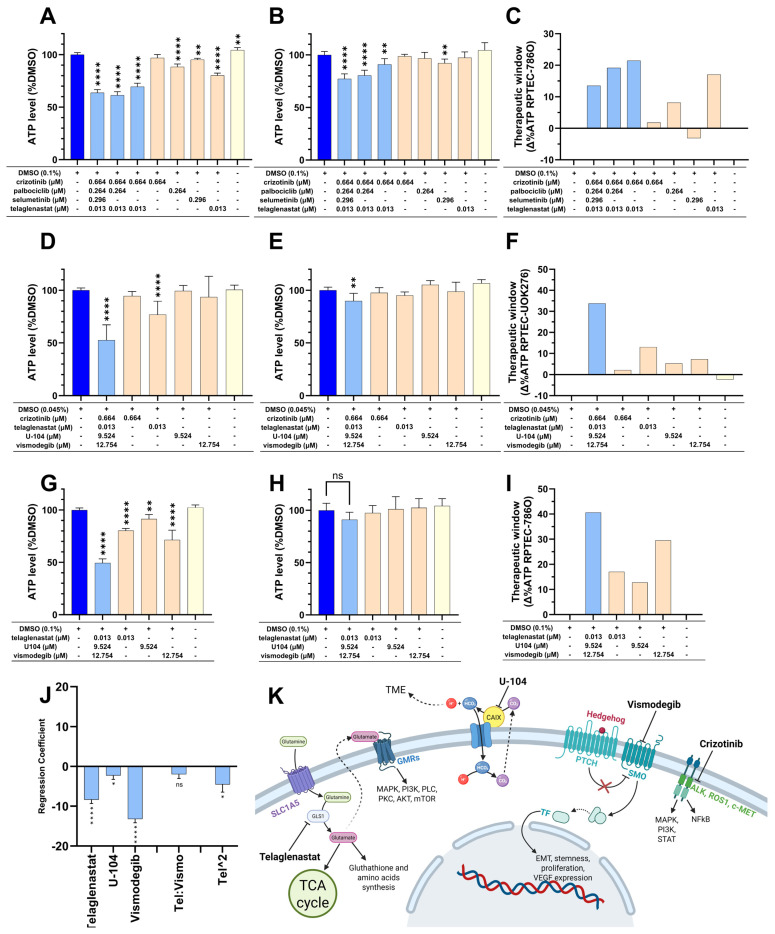
Selectivity of the ODCs. (**A**,**B**) ATP levels of (**A**) 786O cells or (**B**) RPTEC cells treated with variations of the four drugs present in the third search of the 786O-based screen. (**C**) TW of each condition tested in (**A**,**B**) calculated for each condition as the ATP levels of (**A**) subtracted from the ATP levels of (**B**,**D**,**E**) ATP levels of (**D**) UOK276 cells or (**E**) RPTEC cells treated with the four drugs, found in the third search of the UOK276-based screen, combined or as single agents. (**F**) TW of each condition tested in (**D**,**E**) calculated for each condition as the ATP levels of (**D**) subtracted from the ATP levels of (**E**,**G**,**H**) ATP level of (**G**) 786O cells or (**H**) RPTEC cells treated with three drugs of the additive combination found in the 786O-based screen, either combined or as single agents. (**I**) TW of each condition tested in (**G**,**H**) calculated for each condition as the ATP levels of (**G**) subtracted from the ATP levels of (**H**). (**A**,**B**,**D**,**E**,**G**,**H**) Significance was calculated between DMSO controls and all conditions using One-way ANOVA with Dunnett’s multiple comparison test. ^ns^ *p* > 0.05, * *p* < 0.05, ** *p* < 0.01, and **** *p* < 0.0001. (**J**) Regression coefficients estimated from a computational model based on experimental data from treated 786O cells. Histograms are separated by empty ticks into three parts representing (from left to right) 1st order single drug activity, drug–drug interactions, and 2nd order single drug activity. Error bars and stars correspond to the standard deviation and significance of the regression coefficients, respectively. Screening was performed as N = 3 independent experiments. (**K**) Schematic representation of the pathways targeted by the drugs composing the selected ODCs. Created with BioRender.

The effects of these interactions on the cells were confirmed with the CTG data (Figure 1A–C). Consequently, selumetinib and palbociclib were removed for a final combination composed of 0.013 µM telaglenastat and 0.664 µM crizotinib. The 786O-based screen also allowed us to identify a purely additive but effective drug combination (ODC_B_) composed of 0.013 µM telaglenastat, 9.524 µM U-104, and 12.754 µM vismodegib (Figure 1G–I).

In the UOK276-based screen, the last iteration showed only positive interactions for a final combination composed of 0.664 µM crizotinib, 0.013 µM telaglenastat, 9.524 µM U-104, and 12.754 µM vismodegib (Supplementary Figure S8C,D and Figure 1D–F). All three selected combinations will thereon be referred to as ODC_A_, ODC_B_, and ODC_C_ as shown in Table 2.

### 3.2. ODCs Are Effective in Different and Complex Cancer Models but Have Favorable Safety Profiles in Cellular Models Representing Key Organs

**Figure 2:**
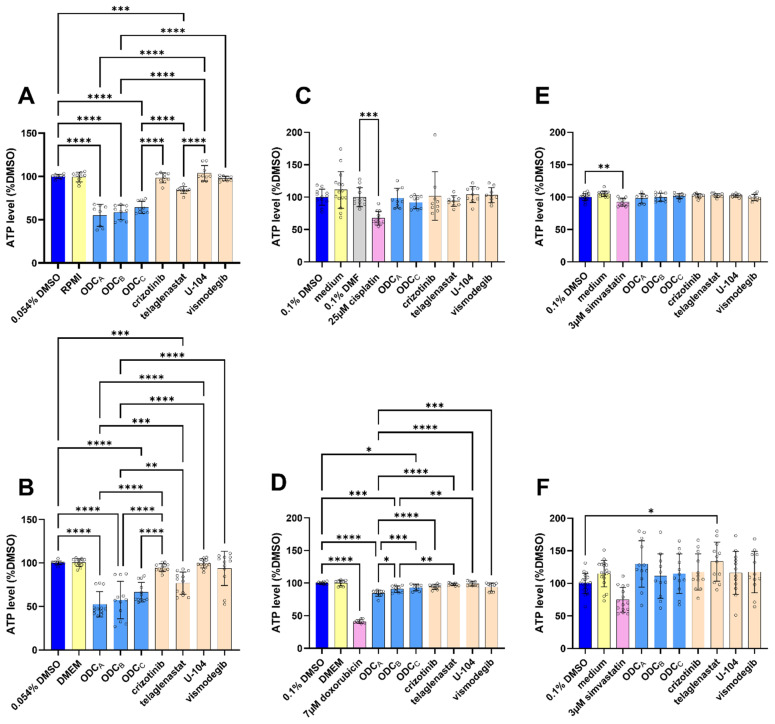
Cross-activity and safety profiles of the ODCs. ATP levels of (**A**) 786O or (**B**) UOK276 cells after 72 h of exposure to the ODCs (light blue) or their composing monotherapies (orange). All data are shown as percentages of the 0.054% DMSO control (dark blue). Culture medium control is presented as a yellow bar. Error bars indicate the standard deviation (metabolic activity measurement, N = 3–4). Circles highlight each technical replicate (n = 2–3). Significance is calculated with One-way ANOVA with Tukey’s multiple comparisons test. (**C**) ATP level of patient-derived kidney organoids (PHK28) treated for 72 h with the ODCs (light blue), corresponding monotherapies (orange), as well as positive (pink) and negative controls (dark blue and yellow). (**D**) ATP level of H9c2 cells differentiated for two weeks with 1 μM all-trans retinoic acid then treated for 72 h with the ODCs (light blue), corresponding monotherapies (orange), as well as positive (pink) and negative controls (dark blue and yellow). (**E**,**F**) ATP level of differentiated HepaRG cells treated for 72 h (**E**) in 2D or (**F**) in 3D with the ODCs (light blue), corresponding monotherapies (orange), as well as positive (pink) and negative controls (dark blue and yellow). (**C**–**F**) All results except (**C**) cisplatin and 0.1% DMF conditions are displayed as a percentage of the 0.1% DMSO control (dark blue). Cisplatin (pink) and 0.1% DMF (grey) conditions are shown as percentages of the 0.1% DMF condition. Significance was calculated with One-way ANOVA with Šídák’s multiple comparisons test. (**A**–**F**) Only comparisons to the DMSO controls, comparisons in-between ODCs, and comparisons between ODCs and their composing monotherapies were kept on the graphs. Significance is displayed as and displayed as * *p* < 0.05, ** *p* < 0.01, *** *p* < 0.001, and **** *p* < 0.0001. The absence of asterisk between mentioned conditions means non-significant differences (*p* > 0.05).

The cross-activity of our ODCs was first investigated. 786O and UOK276 cells were treated with all ODCs and monotherapies (Figure 2A,B). Despite different origins and mutation profiles, both cell lines reacted with similar ATP levels reduction to the different ODCs.

To further assess the safety of our ODCs, they were tested using cellular models of selected healthy organs. We measured the ATP level of renal (Figure 2C), heart (Figure 2D), and liver (Figure 2E,F) models after 72 h of exposure to our ODCs. While various conditions significantly reduced the ATP levels compared to the DMSO control, none of the ODC reduced the viability beyond 80% ATP levels—our threshold for toxicity [38]. This suggests a favorable safety profile in those key organs often affected by xenobiotic toxicity. The absence of toxicity was observed in both proliferative (Supplementary Figure S13A,B) and differentiated (Figure 2D–F) models, suggesting that selectivity is not based on the proliferation rate of the cells.

**Figure 3:**
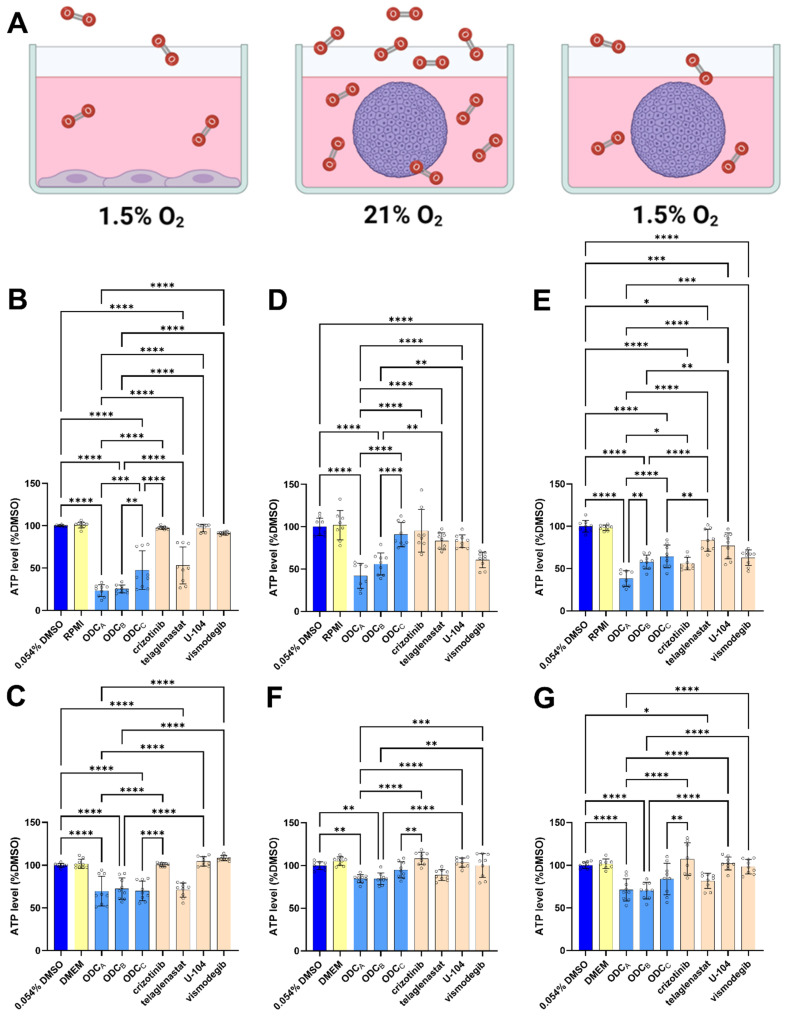
Effect of oxygen and culture dimension on ODCs’ activity. (**A**) Schematic drawing of the different culture/treatment conditions tested in (**B**–**G**). Created with BioRender. (**B**,**C**) ATP levels of (**B**) 786O or (**C**) UOK276 cells cultivated as 2D monolayers in a 1.5% oxygen environment and exposed for 72 h to the ODCs (light blue) or their composing monotherapies (orange). (**D**,**F**) ATP levels of (**D**) 786O and (**F**) UOK276 spheroids cultivated in atmospheric normoxia and exposed for 72 h to the ODCs (light blue) or their composing monotherapies (orange). (**E**,**G**) ATP levels of (**E**) 786O and (**G**) UOK276 spheroids cultivated in a 1.5% oxygen environment and exposed for 72 h to the ODCs (light blue) or their composing monotherapies (orange). (**B**–**G**) All data are shown as percentages of the 0.054% DMSO controls (dark blue). Culture medium control is presented as a yellow bar. Error bars indicate the standard deviation (metabolic activity measurement, N = 3) Circles highlight each technical replicate (n = 2–3). Significance was calculated using One-way ANOVA with Šídák’s multiple comparisons test and displayed as * *p* < 0.05, ** *p* < 0.01, *** *p* < 0.001, and **** *p* < 0.0001. Only comparisons to the DMSO controls, comparisons in-between ODCs, and comparisons between ODCs and their composing monotherapies were kept on the graphs. The absence of an asterisk between mentioned conditions means non-significant differences (*p* > 0.05).

In the next step of ODCs’ efficacy validation, we increased the complexity of our models by incorporating two parameters that can affect the response to treatment, i.e., oxygen concentration and cell culture dimension (Figure 3A). First, we investigated the influence of oxygen on treatment response. Due to poor perfusion, RCC tumors are typically hypoxic (approx. 1.5% oxygen) compared to standard normoxic culture conditions (approx. 21% oxygen) [27]. Under these hypoxic conditions (Figure 3B,C), the effect of the ODCs was potentiated in 786O cells (Figure 3B) but not in UOK276 cells (Figure 3C). In both cases, telaglenastat monotherapy appeared to be the main driver of the effect. In UOK276 cells, it is the only cause of the effect as telaglenastat is the only monotherapy significantly different from the DMSO control and the only condition not significantly different from the three ODCs. In the 786O cells, the response to the ODC_A_ and ODC_B_ seems less reliant on telaglenastat, as they are not affected by the same variations in the independent experiments as the ODC_C_ and the telaglenastat monotherapy.

The second parameter tested was the impact of 3D culture on response to ODCs (Figure 3D–G). 3D cell culture models better replicate in vivo conditions by enabling cellular crosstalk and creating nutrient and oxygen gradients, which more accurately mimic the tumor microenvironment and influence drug response [27,58]. 786O and UOK276 cells were seeded and treated as homotypic spheroids. The effects of the ODCs were evaluated both in normoxia and hypoxia (Figure 3A, middle and right). 786O spheroids in normoxic conditions (Figure 3D) were less affected by ODC_C_ but more by ODC_A_ and ODC_B_. This discrepancy is likely caused by the increased effect of vismodegib, a drug present in ODC_A_ and ODC_B,_ but not in ODC_C_. The efficacy of ODC_C_ is recovered under hypoxic conditions (Figure 3E), along with a potentiated effect of crizotinib.

In UOK276 spheroids (Figure 3F), the effects of all ODCs were reduced compared to 2D. While hypoxia reduced the effect of the ODCs in this cell line in 2D (Figure 3C), in 3D (Figure 3G), part of the effect is increased, driven by a potentiation of the telaglenastat monotherapy.

### 3.3. ODCs Do Not Show Pronounced Activity in Patient-Derived Models

Building on the demonstrated activity of ODCs on distinct RCC subtypes with different mutation profiles, we hypothesized that their mechanism of action might show broad efficacy across various kidney cancer models. To test this hypothesis, we evaluated the ODCs against a panel of patient-derived organoids and spheroids.

**Figure 4:**
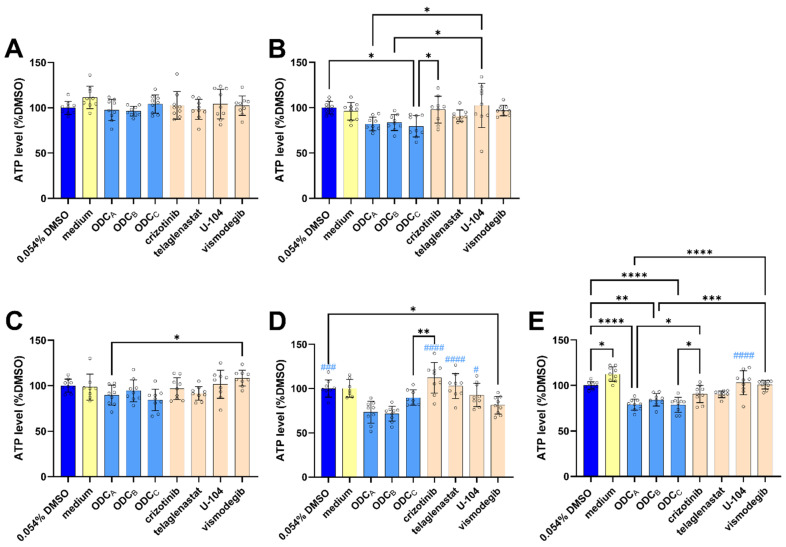
Effect of the ODCs on patient-derived 3D models. ATP levels of (**A**) PRCC1 cancer organoids, (**B**) PRCC20 cancer spheroids, (**C**) PRCC18 cancer spheroids, (**D**) PRCC9 cancer organoids, and (**E**) PRCC17_1 cancer spheroids. For all graphs: 0.054% DMSO control is presented in dark blue, medium control is displayed in yellow, various ODCs are shown in light blue, and monotherapies are presented in orange. Significance was calculated using ordinary one-way ANOVA with Tukey’s multiple comparison test from n = 2–3 technical replicas (displayed as circles in each treatment condition) in N = 3 independent experiments. Significance versus the 0.054% DMSO control, between ODCs, and between ODCs and their composing monotherapies is indicated with * *p* < 0.05, ** *p* < 0.01, *** *p* < 0.001, and **** *p* < 0.0001. Conditions that were significantly different from both ODC_A_ and ODC_B_ were labeled with blue hashtags indicating significance as follows: # *p* < 0.05, ### *p* < 0.001, and #### *p* < 0.0001.

ODCs had variable, but rather disappointing results across the patient samples. PRCC1 organoids (chromophobe RCC, pT2b, Figure 4A) were not affected by any of the treatment conditions. Similarly, for PRCC20 (clear cell RCC, ISUP3 pT3a pN1, Figure 4B), and PRCC18 (papillary RCC, ISUP2 pT1a, Figure 4C), the ODCs exhibited weak to non-significant effects on the viability of the spheroids. Interestingly, ODCs in PRCC9 organoids (clear cell RCC, ISUP3) exhibited reduced overall effects (Figure 4D), and a small but significant activity of the ODCs was observed in PRCC17_1 spheroids (ccRCC, ISUP4, Figure 4E).

While we observed reduced effects compared to 786O spheroids, statistically significant responses occurred exclusively in clear cell RCC samples. This aligns with our previous observations of superior ODC sensitivity in 786O (ccRCC) cells when compared to UOK276 (chRCC) cells in 3D models. This, in turn, highlights the need for molecular stratification to identify likely responders and potential non-responders.

### 3.4. Chronic Treatment with ODCs Does Not Induce Acquired Resistance in Cells

**Figure 5:**
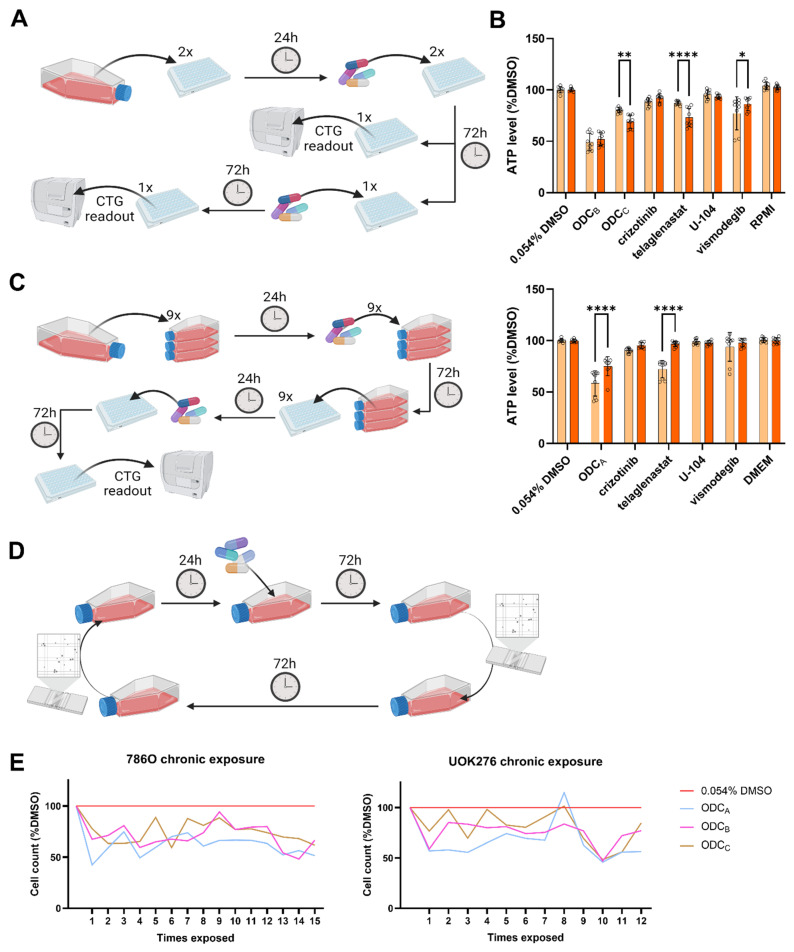
Effect of repeated exposure on treatment response. (**A**,**C**,**D**) Schematic representation of the different re-treatment strategies adopted for retreatment of (**A**) the same plate, (**C**) the same cell population, and (**D**) of the same cell population for a long period. (**B**) Response to first (orange) and second (red) treatment in 786O (top) and UOK276 (bottom) following the method described in (**A**). Each cell line was treated only with the ODC(s) and corresponding monotherapies identified in that same cell line. Both graphs are the results of N = 3 independent experiments including n = 3 technical replicate for each condition. (**C**) Data generated following this protocol can be found in Supplementary Figures S14 and S15. (**E**) Results of the retreatment with ODC conducted as indicated in (**D**) on 786O (left) and UOK276 (right) cells. Each point in the graphs corresponds to the mean of three measurement from N = 1 independent experiment. Color code can be found in the legend (far right) and is valid for both cell lines. Graduation on the *x*-axis corresponds to the number of times the cells were exposed to the drugs for 72 h. Circles highlight each technical replicate (n = 3). Significance was calculated using Two-way ANOVA with Šídák’s multiple comparisons test and displayed as * *p* < 0.05, ** *p* < 0.01, and **** *p* < 0.0001.

It is known that repeated exposure to a treatment can modulate the cellular response, potentially resulting in sensitization or resistance of the cells [11]. To evaluate this effect in our ODCs, we first investigated by treating the same 96-well plate twice for 72 h (Figure 5A). After the second ODC_A_ treatment, UOK276 cells exhibited reduced ATP levels, directly linked to the effect of telaglenastat monotherapy (Figure 5B). In 786O cells, the ODC_C_ showed a minor decrease in ATP levels after retreatment, linked as well to a decreased activity of telaglenastat monotherapy. 786O cells treated with the ODC_B_ showed similar ATP levels after the first and second treatments. In this case, the reduction of ATP levels induced by the telaglenastat monotherapy may have been counteracted by the reduced effect of the vismodegib monotherapy.

While the initial retreatment approach enabled the assessment of cumulative drug effects on the same well plate of cells, extended culture duration in a static container introduced confounding factors. Specifically, vehicle control normalization may become problematic as control wells grow overconfluent. To address this limitation, we implemented a modified protocol where cells received an initial 72-h treatment in a culture flask, under optimal growth conditions, followed by seeding and treatment in 96-well plates (Figure 5C). This new design maintains the advantages of retreatment analysis while minimizing confluency-related artifacts in control measurements. The sole condition in which pre-exposure induced a significant change in treatment response was the condition where cells treated with telaglenastat presented lower levels of ATP when they were pre-exposed to ODC_C_ (Supplementary Figure S14). All other conditions remained unaffected. Analysis of UOK276 cells (Supplementary Figure S15) revealed diminished response to all ODCs during retreatment cycles. This could be due to reduced activity of telaglenastat under comparable conditions. Similar, but non-significant, trends are observed in cells pre-exposed to telaglenastat monotherapy.

To characterize the temporal evolution of treatment response, we extended our analysis through weekly retreatment cycles (Figure 5D). While we observed experimental inter-treatment variations, the reduction in cell population induced by the ODC_A_ compared to the 0.045% DMSO control stabilized around 50–60% for both cell lines (Figure 5E). Similar trends were observed with the ODC_B_ and ODC_C_ at slightly higher viability rates. This durability suggests potential clinical relevance of chronic administration regimens.

### 3.5. Cell Cycle Arrest and Cell Death Induction Play Partial Roles in the Mechanism of Action of ODCs

**Figure 6:**
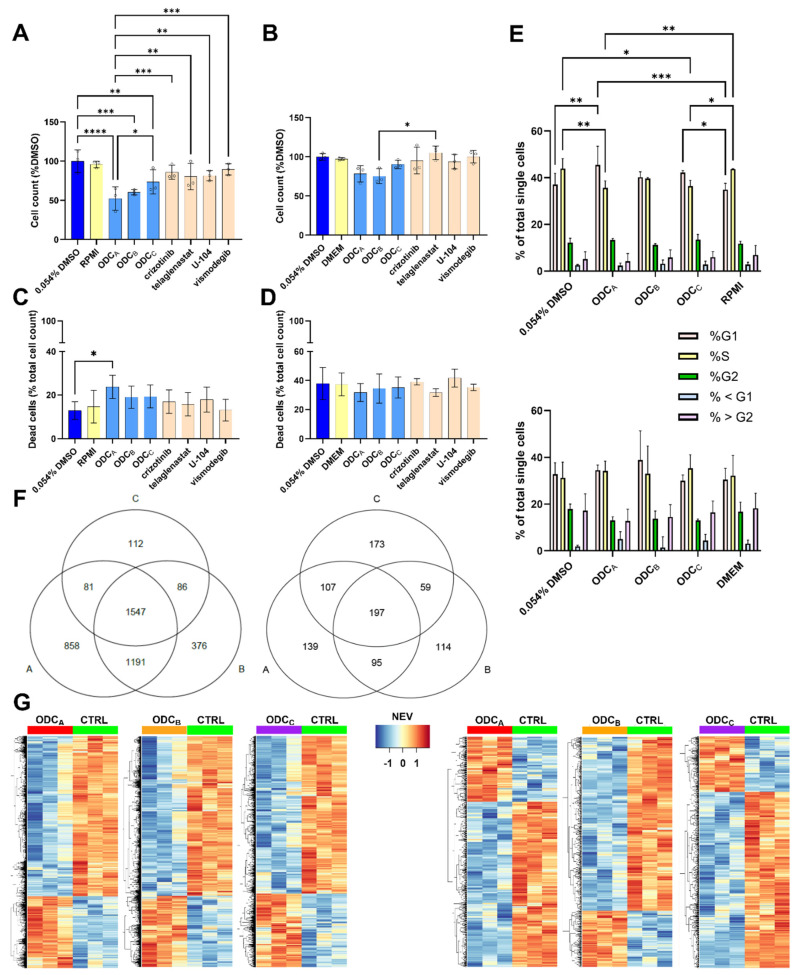
Insight into the mechanism of the ODCs. Percentage of counted living cells in treated (**A**) 786O and (**B**) UOK276 cells compared to the count in the 0.054% DMSO control. (**C**,**D**) Percentage of dead cells compared to the total cell count in each treated condition for (**C**) 786O-treated flasks and (**D**) UOK276-treated flasks. (**A**–**D**) Error bars indicate the standard deviation (cell count measurement of N = 3 independent experiments). Circles highlight each technical replicate (n = 1). Significance was calculated using Two-way ANOVA with Tukey’s multiple comparisons test and displayed as * *p* < 0.05, ** *p* < 0.01, *** *p* < 0.001, and **** *p* < 0.0001. (**E**) Cell cycle distribution of 786O (top) and UOK276 (bottom) cells exposed for 24 h to the ODCs or controls. The cell cycle phases color code in both graphs is as indicated in the legend (middle). Significance was calculated between similar cell cycle phases of all conditions using Two-way ANOVA with Tukey’s multiple comparisons test and displayed as * *p* < 0.05, ** *p* < 0.01, and *** *p* < 0.001. Examples of the gating can be found in Supplementary Figure S17. (**F**) Venn diagram of genes significantly affected by ODC_A_ (A), ODC_B_ (B), and/or ODC_C_ (C) in 786O (left) and UOK276 (right) cells. (**G**) Heatmaps of up- (red) and down- (blue) regulated genes in 786O (left) and UOK276 (right) cells after 72 h of treatment with the ODCs. “NEV” (center) stands for Normalized Expression Values. (**F**,**G**) Only the genes that are significantly different (*p* < 0.05) and have a fold change superior to or equal to 2 compared to the 0.054% DMSO control are displayed.

To verify whether the measured ATP levels are correctly correlated to the number of cells, the cell viability was assessed with an orthogonal method, in which treated cells were harvested and counted using trypan blue exclusion dye. In 786O cells (Figure 6A), the measured cell counts gave similar results to the CTG readout, confirming the previously observed decrease in cell viability. However, in the UOK276 cells (Figure 6B), the decrease in cell number did not correspond to the ATP levels of the CTG readout. This suggests that the response to the ODCs observed on the UOK276 cells using CTG could be, at least partially, caused by metabolic changes.

The trypan blue exclusion cell counting assay (Figure 6C,D) served as an orthogonal method to verify the CTG results while providing quantitative cytotoxic data after treatment. In 786O cells, only ODC_A_ treatment demonstrated a statistically significant increase in dead cell percentage compared to the control. In UOK276 cells, the percentage of dead cells remained consistent across all tested conditions. Likewise, in the 786O cells, cytotoxicity was unlikely to be the main driver of the activity. This was further supported by a cell death assay, in which no difference in the percentage of cells in early apoptosis/late apoptosis/necrosis was observed across all drug combinations (Supplementary Figure S16A,B).

To assess treatment-induced cell cycle perturbations, we performed flow cytometry analysis of PI-stained cells following 24-h drug exposure. This approach enabled quantitative determination of cell cycle phase distribution across experimental conditions.

The results (Figure 6E) showed a moderate increase in the G1 phase and a decrease in the S phase for ODC_A_. ODC_C_ showed similar significant effects compared to medium control, but only a decrease in the S phase compared to the 0.054% DMSO control. Cell death (% < G1 and % > G2) was not predominant and was similar throughout all conditions. In UOK276 cells, none of the ODCs caused significant modification to the cell cycle distribution. This suggests common components in ODC_A_ and ODC_C_ may induce cell cycle arrest in 786O but not in UOK276 cells.

To further investigate the mechanisms of action, we performed bulk RNA sequencing on cells exposed to the ODCs for 72 h (Figure 6F,G). The analysis showed more pronounced transcriptional changes in 786O cells than in UOK276 cells when comparing combinations to controls/monotherapies. Significant overlaps were observed in affected genes across ODCs, which is consistent with their shared drug composition (Figure 6F). Although the gene expression profiles of the cells treated with the monotherapies were extremely close to one of the controls (Supplementary Figures S17 and S21), the addition of a single drug into a combination had a substantial impact on gene expression. For instance, in 786O cells, the addition of crizotinib in ODC_A_ compared to ODC_B_ significantly affected the expression of 858 genes (Figure 6F). Compared to the DMSO control, upregulated genes in 786O cells treated with either ODC were principally linked to cholesterol synthesis, lipid metabolism, and inflammatory response, whereas downregulated genes grouped in rather undefined clusters with no significant adjusted *p*-value (Supplementary Figures S18–S20, Tables S1–S6).

In contrast, pathways associated with upregulated genes, in UOK276 cells treated with ODC_A_, had non-significant adjusted *p*-values, and pathways associated with downregulated genes showed significance. Most of those genes are related to the extracellular matrix (Supplementary Figures S22–S24, Tables S7–S12).

## 4. Discussion

Using our set of ten drugs and two human RCC cell lines, we performed the TGMO-based screen, which allowed in only three experimental searches, the identification of three optimized drug combinations. Two of them (ODC_A_ and ODC_C_) were selected based on the presence of positive and absence of pejorative drug–drug interactions. ODC_B_ was selected based on the individual effects of the drug and the absence of negative drug–drug interactions.

In our screen, particular attention was given to safety. Osimertinib, which was initially central to the screen, was rapidly excluded in search 1 due to its high toxicity in the RPTEC model. Furthermore, none of the ODC-composing drugs were dosed higher than the mean plasma concentration in patients. For example, the doses of telaglenastat and vismodegib were 169-fold and 1.3-fold lower than the CUD (Table 1 and Table 2). While this strategy was successful as all ODCs exhibited favorable safety profiles in healthy organ models (Figure 1B,E,H and Figure 2C–F and Supplementary Figure S13), it certainly affected the final antineoplastic effect of our combinations compared to previous TGMO-based ODCs [13]. Moreover, our initial drug set, which was mostly based on theoretical assumptions, proved suboptimal, as most compounds, particularly in UOK276 cells, exhibited IC_20_ values far exceeding CUD (Table 1; Supplementary Figure S1).

While individual drugs from the initial set showed limited individual efficacy, the overall outcome is encouraging and supports the use of the TGMO approach through two key findings. First, the three most effective single agents at CUD (Supplementary Figure S1) demonstrated no antagonistic interactions in 786O cells and exhibited positive interactions in UOK276 cells (Figure 1J; Supplementary Figure S8D). This is encouraging as additive combinations are suspected to better retain their effect on different models [18]. Second, GLS1 inhibitor telaglenastat demonstrated positive interactions with MET inhibitor crizotinib in both RCC models (Supplementary Figures S5D and S8D). The latter interaction is supported by another study showing positive interactions between telaglenastat and another MET inhibitor cabozantinib [59]. Moreover, since our novel ODCs exhibited no signs of toxicity in the tested models, subsequent screens could be performed to incorporate additional drug(s) with controlled risks to safety. However, caution is warranted, as adding more drugs may increase the risks of unforeseen interactions, as well as complicate the administration [21,38,60]. Alternatively, our ODCs could be used alone as a later line of treatment where the goal shifts from curing to managing the tumor with minimal effect on the quality of life of the patient [61]. Furthermore, the apparent absence of resistance after several months of treatment (Figure 5E) supports the potential use of the combinations as a long-term adjuvant to other therapies.

Contrary to expectations, where drug activity typically diminished in more complex models [27], our combination therapy demonstrated enhanced performance under tumor-relevant conditions, i.e., in the presence of hypoxia and using 3D cellular models (Figure 3B,C). For instance, hypoxic conditions that are commonly found in tumors can, among others, influence cell metabolism, targeted protein expression, and activity of compensatory pathways, as well as the expression of efflux pumps—various conditions that can reduce the exposure or effectiveness of a drug [62,63]. The enhanced efficacy of our ODCs in hypoxia-cultured 786O cells was particularly surprising, given their inherent pseudo-hypoxic phenotype resulting from VHL gene inactivation [27,64]. Moreover, 786O cells lack functional HIF-1α expression resulting in a partial, HIF-2α-mediated, hypoxic response [64,65,66]. For instance, while HIF-2α can influence glutamine metabolism reprogramming, its role is mostly limited to pro-angiogenic response and stem phenotype induction, while HIF-1α is more active in cell metabolism [67,68,69,70]. The observed enhancement of ODCs efficacy under hypoxia presents a paradox in 786O cells, given their established pseudo-hypoxic phenotype [71].

In UOK276 cells, which should retain a functional VHL response [30], the decrease in ATP levels induced by the ODCs was comparable to telaglenastat. Although telaglenastat showed enhanced effects in both cell lines, which is consistent with the increased importance of its target (GLS1) in hypoxic cells [25], the more consistent response to ODC_A_ and ODC_B_ in 786O cells suggests a greater implication of U-104 and vismodegib in this model.

Three-dimensional cellular models better mimic the in vivo tumor microenvironment in terms of nutrient gradients, oxygen levels, access to therapeutic agents, and increased cellular crosstalk [27,58]. These parameters make it more physiologically relevant and may contribute to drug resistance in vitro [27,72]. While in UOK276 spheroids, ODCs demonstrated a ubiquitous activity reduction; the response in 786O spheroids was rather variable (Figure 3D–F). Compared to 786O cells in 2D, the ODC_A_ showed a superior effect, the ODC_B_ retained similar potency, and ODC_C_ lost its activity. Interestingly, the reduced activity observed with the ODC_C_ was specifically attributable to the combination itself, rather than to any decrease in efficacy of its component, e.g., crizotinib or telaglenastat. No significant changes in activity were detected for either drug when administered alone. The increased and sustained effect observed with ODC_A_ and ODC_B_ could be attributed to the increase in the potency of vismodegib and U-104. While telaglenastat exhibited increased efficacy under 2D hypoxic conditions in both cell lines, the potentiation of vismodegib and U-104 in 3D cultures was observed exclusively in the 786O spheroids. Notably, similarly to the 2D models, treatment under hypoxia amplified the response to ODCs. However, in this case, telaglenastat was involved in this enhanced response only in UOK276 spheroids, whereas in 786O spheroids, the increased response was mostly mediated by crizotinib. Currently available data do not allow for a strong hypothesis concerning the mechanism of action behind these observations.

By simultaneously targeting multiple pathways, the ODCs may address two key challenges in RCC treatment, i.e., promote a more homogenous response in this heterogeneous tumor type [27], and delay resistance emergence. Although not all patients could benefit from these ODCs, as demonstrated by the limited response in UOK276 cells and patient-derived models (Figure 4), the 786O data, showing sustained ODC efficacy mediated by alternate drugs across conditions, strongly advocate in favor of the use of drug combinations. Moreover, the increased ODCs efficacy under hypoxia suggests improved and particularly relevant tumor selectivity, given that RCC tumors exhibit median oxygen levels four-fold lower than healthy kidney tissue [27].

While initially all ODCs appeared to affect both cell lines alike (Figure 2A,B), further experiments showed their limited short-term effects in UOK276 cells (Figure 3 and Figure 6). We hypothesize that this response discrepancy could be mediated by changes in the UOK276 cell metabolism affecting the CTG readout. Indeed, a decrease in cellular ATP production would align with a telaglenastat-induced inhibition of glutaminolysis [25,73].

Increased biosynthesis of cholesterol observed in all RNAseq data may also be explained by the mechanism of action of telaglenastat. Indeed, glutamine is an important regulator of cholesterol synthesis in cells [74]. By inhibiting GLS1, telaglenastat may increase the cellular levels of glutamine, thus triggering the mevalonate pathway [59,74]. Whether this increase in cholesterol is a compensatory mechanism, a byproduct, or intervenes in the mechanism of action of the ODC remains to be elucidated.

The results obtained with the chromophobe cell line UOK276 highlight two key challenges in RCC treatment. First, necessity for orthogonal viability assessment methods, especially for assays based on cellular metabolic activity. Second, the limitations of current one-size-fits-all therapeutic approaches for both ccRCC and chRCC/sarcomatoid subtypes. While our initial drug selection or readout method may have been suboptimal for UOK276 cells, our results strongly support the value of systematic drug combination optimization platforms like the TGMO. Such tools enable either the development of personalized treatments for stratified patient subgroups or the identification of broadly effective combinations across subtypes.

To conclude, the TGMO approach allowed the identification of three multidrug combinations (ODC_A_, ODC_B_, and ODC_C_) composed of crizotinib, telaglenastat, U-104, and vismodegib. While the efficacy of these drug combinations in non-clear cell RCC models showed suboptimal efficacy in cell line models, they exhibited several advantageous features worth further investigation. Firstly, the identified ODCs demonstrated no detectable toxicity in complex in vitro models of key organs, confirming a favourable preliminary safety profile. Furthermore, the drug combinations retained consistent activity following chronic drug exposure and demonstrated improved activity in tumor-relevant hypoxic conditions and in 3D spheroid models of the 786O cell line. Collectively, these preclinical observations underscore the value of the TGMO approach for drug discovery and provide a rationale for future in vitro studies to evaluate their therapeutic potential.

## Figures and Tables

**Table 1:** Clinically used doses (CUD) of the drugs selected in our TGMO-based screen set. All CUDs are given in µM [48,49,50,51,52,53,54,55,56,57].

Drug	Aspirin	Axitinib	Crizotinib	Osimertinib	Palbociclib	Selumetinib	Simvastatin	Telaglenastat	U104	Vismodegib
CUD (µM)	80,000	0.029	0.664	0.469	0.264	0.296	0.005	2.113	9.524	17,000 *

* indicates that the CUD was not calculated from the AUC but from the median plasma concentration at steady state.

**Table 2:** Summary of the optimized drug combinations (ODC) obtained from the TGMO-based screens.

	ODC_A_ _Screened in UOK276 Cells_	ODC_B_ _Screened in 786O Cells_	ODC_C_ _Screened in 786O Cells_
Crizotinib (µM)	0.664		0.664
Telaglenastat (µM)	0.013	0.013	0.013
U-104 (µM)	9.524	9.524	
Vismodegib (µM)	12.754	12.754	

## Data Availability

The datasets used and/or analysed during the current study are available from the corresponding author on reasonable request.

## Supplementary Materials

The following supporting information can be downloaded at: https://www.mdpi.com/article/10.3390/pharmaceutics17101269/s1, Figure S1: (A) Dose-response curve for the 10 drugs included in the TGMO screening pool. (B) Calculation of the IC20 of each drug based on the equation of the curves in (A); Figure S2: Sorted experimental measurements of the TGMO-based search 1 screen on RPTEC cells; Figure S3: Sorted experimental measurements of searches 1–3 of the TGMO-based screen on 786O cells; Figure S4: Sorted experimental measurements of searches 1–3 of the TGMO-based screen on RPTEC cells as part of the 786O TGMO-based screening; Figure S5: Searches 1–3 of the TGMO-based screen in the 786O cells and RPTEC cells; Figure S6: Sorted experimental measurements of searches 1–3 of the TGMO-based screen on UOK276 cells; Figure S7: Sorted experimental measurements of searches 2 and 3 of the TGMO-based screen in RPTEC cells as part of the UOK276 TGMO-based screening; Figure S8: Searches 1–3 of the TGMO-based screen in the UOK276 cells and RPTEC cells; Figure S9: Statistical output of the linear regression models to interpret the TGMO-based search in (A) 786O cells search 1, (B) UOK276 cells search 1, (C) TW of the 786O search 1, and (D) TW of the UOK276 search 1; Figure S10: Statistical output of the linear regression models to interpret the TGMO-based search in (A) 786O cells search 2, (B) UOK276 cells search 2, (C) TW of the 786O search 2, and (D) TW of the UOK276 search 2; Figure S11: Statistical output of the linear regression models to interpret the TGMO-based search in (A) 786O cells search 3, (B) UOK276 cells search 3, (C) TW of the 786O search 3, and (D) TW of the UOK276 search 3 all from the screen with higher doses of the drugs; Figure S12: Statistical output of the linear regression models to interpret the TGMO-based search in (A) 786O cells search 3, (B) UOK cells search 3, (C) TW of the 786O search 3, and (D) TW of the UOK276 search 3 all from the screen with original doses of the drugs; Figure S13: Safety profile of the ODCs in non-differentiated or semi-differentiated models; Figure S14: Effect of re-treatment on 786O cells; Figure S15: Effect of re-treatment on UOK276 cells; Figure S16: Cell death assay analysis on treated 786-O (A) and UOK276 (B) cells; Figure S17: Gating procedure used for the cell cycle analysis; Figure S18: Heatmap of the top 500 most variable genes across samples in treated 786O cells corrected for batch effect; Figures S19–S21: Pathways linked to the changes observed in the RNA sequencing data of 786O cells treated with ODC_A_, ODC_B_, and ODC_C_; Figure S22: Heatmap of the top 500 most variable genes across samples in treated UOK276 cells corrected for batch effect; Figures S23–S25: Pathways linked to the changes observed in the RNA sequencing data of UOK276 cells treated with ODC_A_, ODC_B_, and ODC_C_; Tables S1–S6: Pathways linked to the changes observed in the RNA sequencing data of 786O cells treated with ODC_A_, ODC_B_, and ODC_C_; Tables S7–S12: Pathways linked to the changes observed in the RNA sequencing data of UOK276 cells treated with ODC_A_, ODC_B_, and ODC_C_.

## Abbreviations

ccRCC	clear cell renal cell carcinoma
chRCC	chromophobe renal cell carcinoma
CTG	CellTiter-Glo^®^
CUD	clinically used dose
ODC	optimized drug combination
PI	propidium iodide
RCC	renal cell carcinoma
TGMO	therapeutically guided multidrug optimization
TKI	tyrosine kinase inhibitors
TW	therapeutic window
VEGFR	vascular endothelial growth factor receptors
