# Supplementary material for: Rational Design of Non-Toxic Multidrug Combinations Demonstrates Durable and Hypoxia-Enhanced Efficacy Against Renal Cell Carcinoma
Source: Pharmaceutics. 2025 Sep 27;17(10):1269. doi: 10.3390/pharmaceutics17101269 (PMC12567191; doi:10.3390/pharmaceutics17101269)
Supplement: Supplementary file 1 [file pharmaceutics-17-01269-s001.zip › pharmaceutics-3803898-supplementary.pdf]

Supplementary Material to:

Rational Design of Non-Toxic Multidrug Combinations Demonstrates Durable and Hypoxia-Enhanced Efficacy Against Renal Cell Carcinoma

Valentin Miéville[1,2,3], Jakub Gubala[1,2,3], Mathis Fiault[1,2,3], Marie Ota[1,2], Seungsu Han[1,2], Muriel Urwyler[1,2,3], Daniel Benamran[4], Jean-Christophe Tille[5], Massimo Valerio[4], Patrycja Nowak-Sliwinska[1,2,3] *

[1] School of Pharmaceutical Sciences, Faculty of Science, University of Geneva, 1211 Geneva, Switzerland

[2] Institute of Pharmaceutical Sciences of Western Switzerland, University of Geneva, 1211 Geneva, Switzerland

[3] Translational Research Center in Oncohaematology, 1211 Geneva, Switzerland

[4] Division of Urology, Geneva University Hospitals, 1211 Geneva, Switzerland

[5] Division of Pathology, Geneva University Hospitals, 1205 Geneva, Switzerland

A

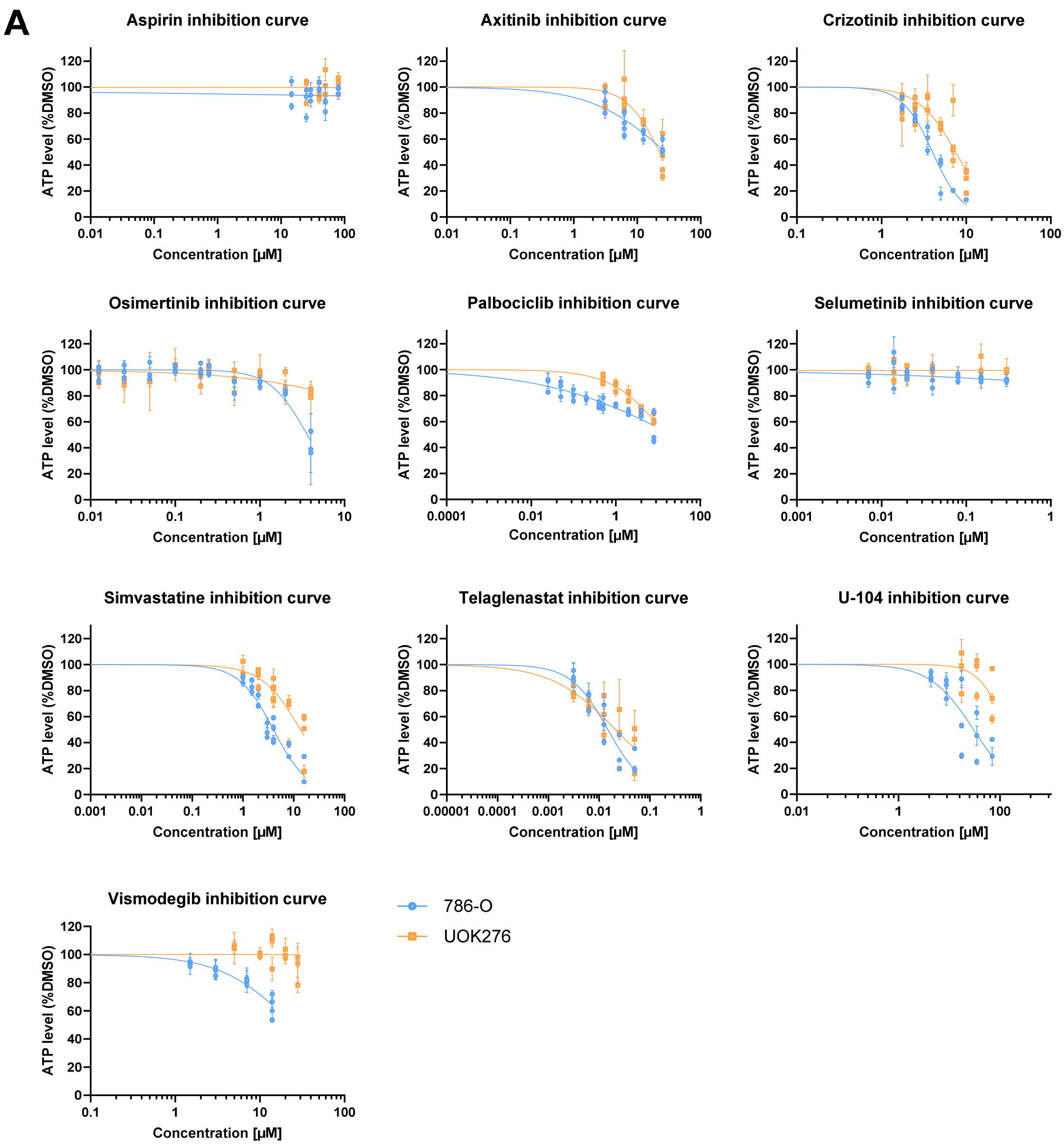

B

log(inhibitor) vs. normalized response -- Variable slope	
Y=	100/(1+10^((LogIC50-X)*HillSlope)))
Z=	(1+10^A)
A=	log((LogIC50-X)*Hill! Pour Y=80, A= -0.60205999
X=	.-A/HillSlope+logIC50

Log(IC50)	raw data provided in prism Dose response curve results
HillSlope	raw data provided in prism Dose response curve results

	Axitinib		Crizotinib		Osimertinib		Palbociclib		Simvastatin		Telaglenastat		U-104		Vismodegib	
	786O	UOK276	786O	UOK276	786O	UOK276	786O	UOK276	786O	UOK276	786O	UOK276	786O	UOK276	786O	UOK276
A	-0.60206	-0.60206	-0.60206	-0.60206	-0.60206	-0.60206	-0.60206	-0.60206	-0.60206	-0.60206	-0.60206	-0.60206	-0.60206	-0.60206	-0.60206	-0.60206
Log(IC50)	1.447	1.35	0.6023	0.8761	0.5589	2.019	1.33	1.177	0.6003	1.132	-1.828	-1.68	1.475	2.12	1.392	1.468
HillSlope	-0.7352	-1.67	-2.437	-1.931	-1.998	-0.524	-0.2833	-0.6989	-1.272	-1.133	-1.194	-0.6908	-1.026	-1.644	-1.025	Unstable
X	0.6281	0.9895	0.3553	0.5643	0.2576	0.8700	-0.7952	0.3156	0.1270	0.6006	-2.3322	-2.5515	0.8882	1.7538	0.8046	NA
**IC20 (µM)**	**4.2471**	**9.7608**	**2.2660**	**3.6670**	**1.8095**	**7.4136**	**0.1603**	**2.0680**	**1.3396**	**3.9867**	**0.0047**	**0.0028**	**7.7303**	**56.7262**	**6.3771**	**NA**

Supplementary Figure S1. A) Dose-response curve for the 10 drugs included in the TGMO screening pool. Drug responses were performed in 786O (blue) and UOK276 (orange) cells treated for 72 hours. Error bars correspond to the standard deviation of N=3-5 independent experiments. Continuous lines display the GraphPad Prism® embedded analysis "*non-linear fit curve (log(inhibitor) vs normalized response – Variable slope*". **B**) Calculation of the IC_{20} of each drug based on the equation of the curves in **A**).

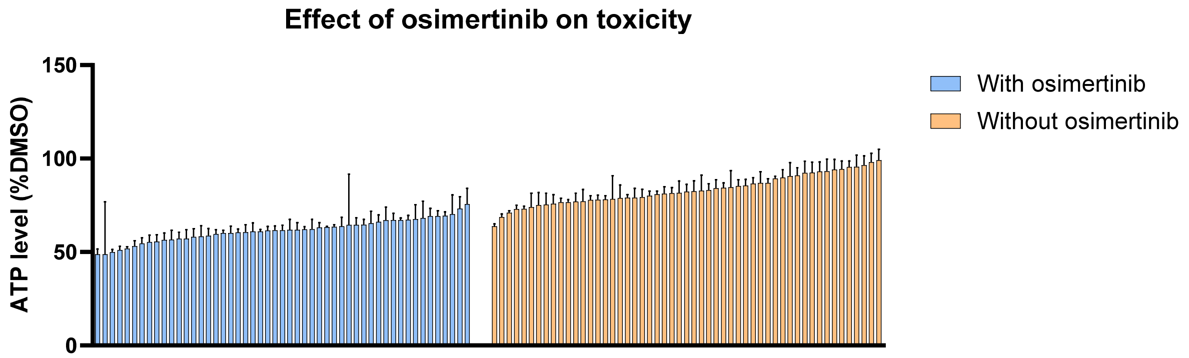

Supplementary Figure S2. Sorted experimental measurements of the TGMO-based search 1 screen on RPTEC cells. Measured ATP values normalized as a percentage of the DMSO vehicle control were sorted into two categories: treatment conditions that contain osimertinib (blue) and treatment conditions that are free of osimertinib (orange). Control conditions that do not contain any drug were not included. In both categories, conditions were sorted from most to least active. Bars correspond to the standard deviation of 3 technical replicates from 1 independent experiment.

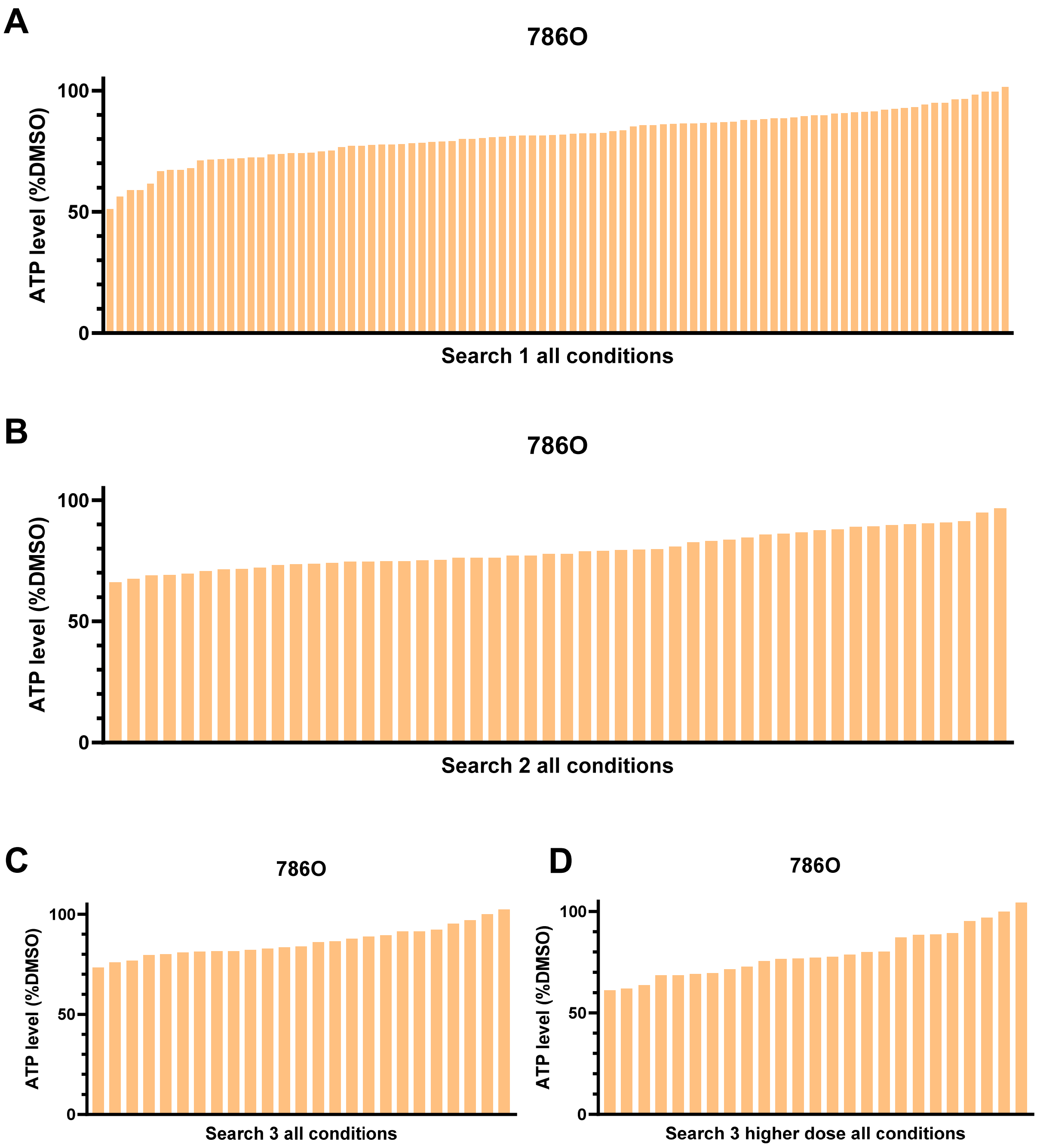

Supplementary Figure S3. Sorted experimental measurements of searches 1-3 of the TGMO-based screen on 786O cells. Sorted ATP level averages from lowest to highest of all conditions tested in searches **A**) 1, **B**) 2, and **C,D**) 3 of the TGMO-based screening on 786O cells.

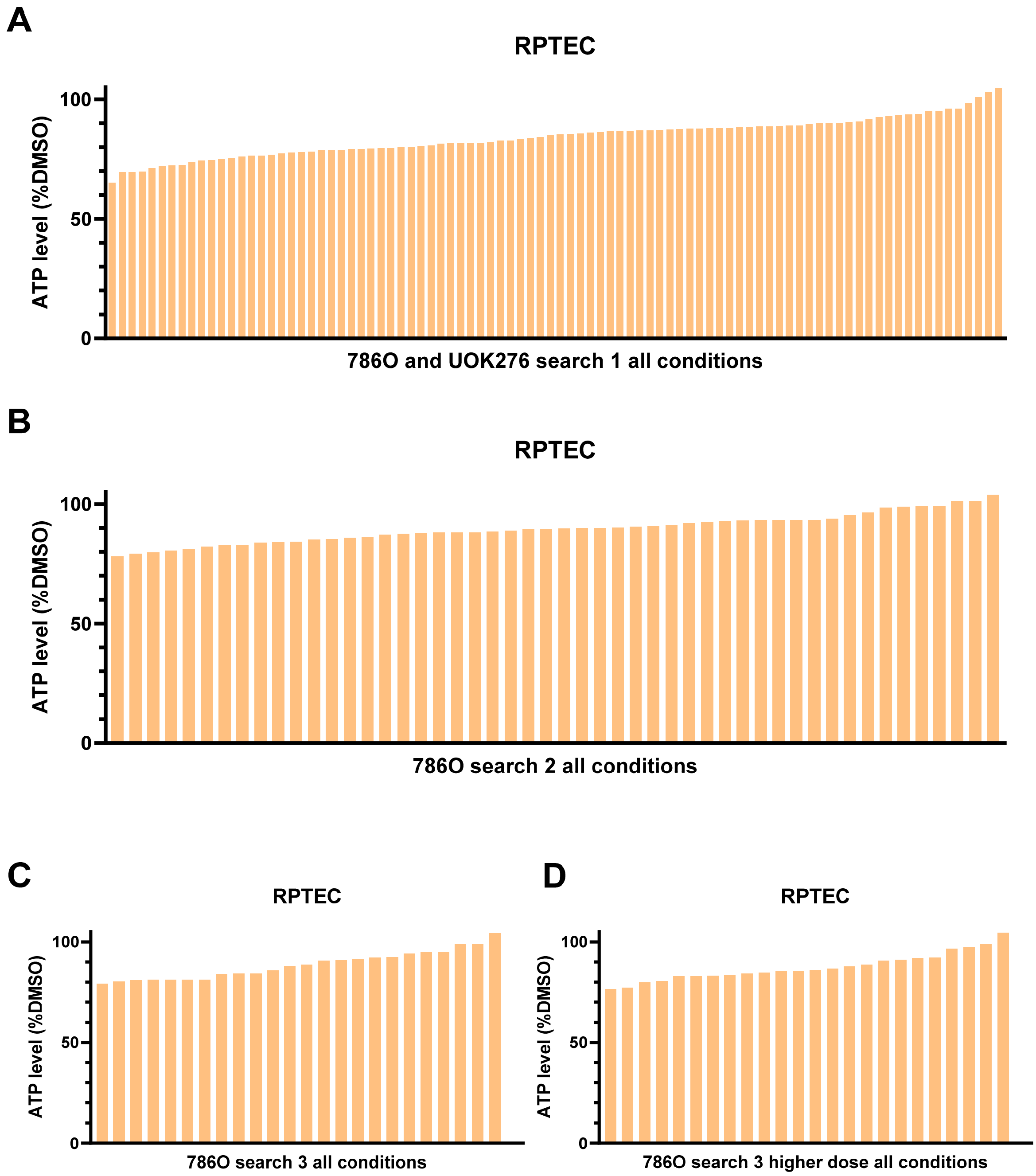

Supplementary Figure S4. Sorted experimental measurements of searches 1-3 of the TGMO-based screen on RPTEC cells as part of the 786O TGMO-based screening. Sorted ATP level averages from lowest to highest of all conditions tested in RPTEC cells in iterations **A**) 1, **B**) 2, and **C,D**) 3 of the TGMO screening on 786O cells. **A**) is shared for both 786O and UOK276 screens.

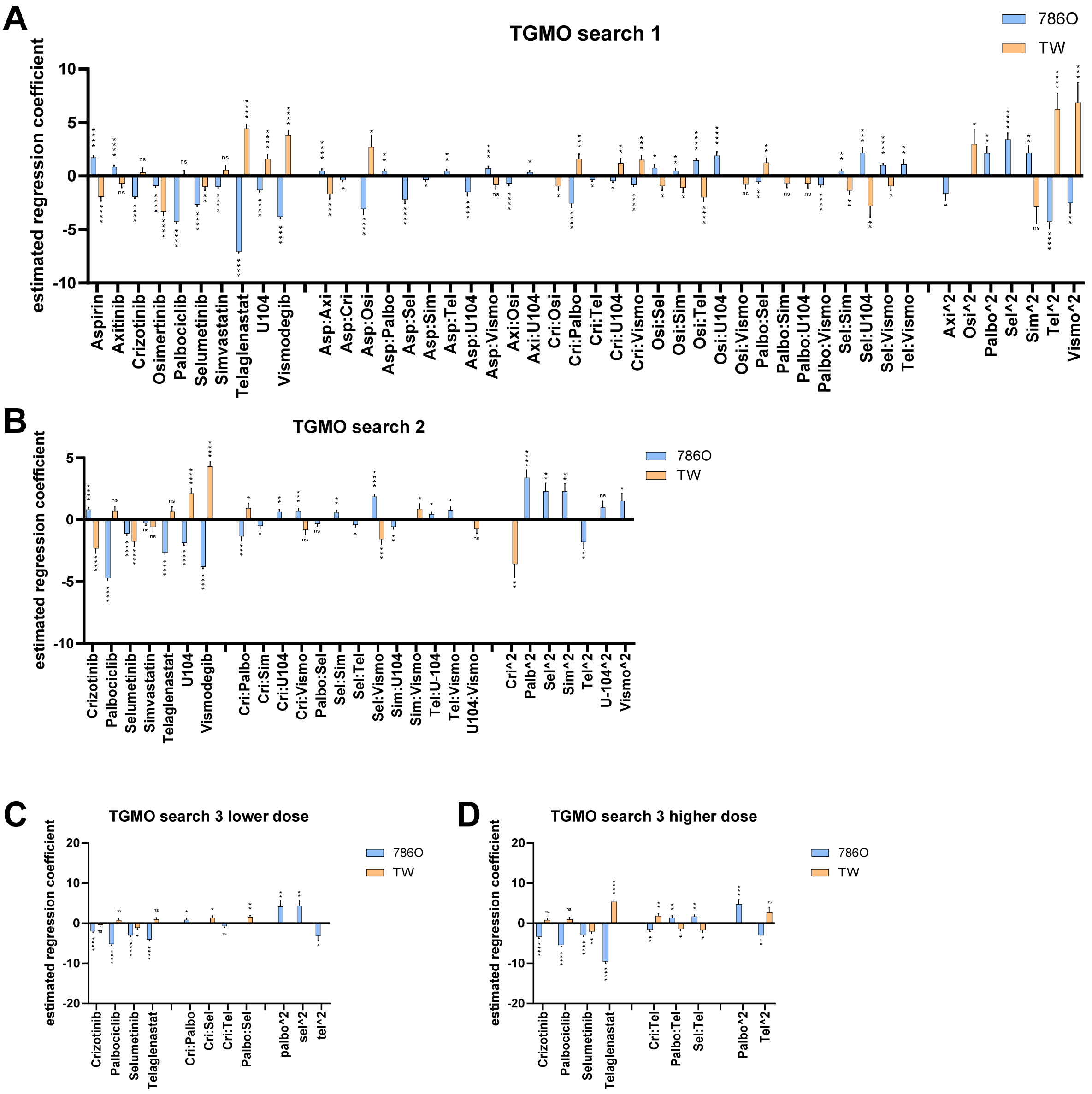

Supplementary Figure S5. Searches 1-3 of the TGMO-based screen in the 786O cells and RPTEC cells. Regression coefficients are estimated from a computational model based on experimental data input of the **A**) search 1, **B**) search 2, and **C,D**) search 3. Parts of the histograms are separated by empty thick into three parts representing (from left to right) 1st order single drug activity, drug-drug interactions, and 2nd order single drug activity. Graphs display the effect on 786O cells (blue) and therapeutic window (TW) – calculated as the difference between the experimental effect on healthy RPTEC cells and cancerous 786O cells. Error bars and stars respectively correspond to the standard deviation and significance of the estimated regression coefficients. All screenings were performed as N=3 independent experiments. Abbreviations: Aspirin (Asp), axitinib (Axi), crizotinib (Cri), osimertinib (Osi), palbociclib (Palbo), selumetinib (Sel), simvastatin (Sim), telaglenastat (Tel), vismodegib (Vismo).

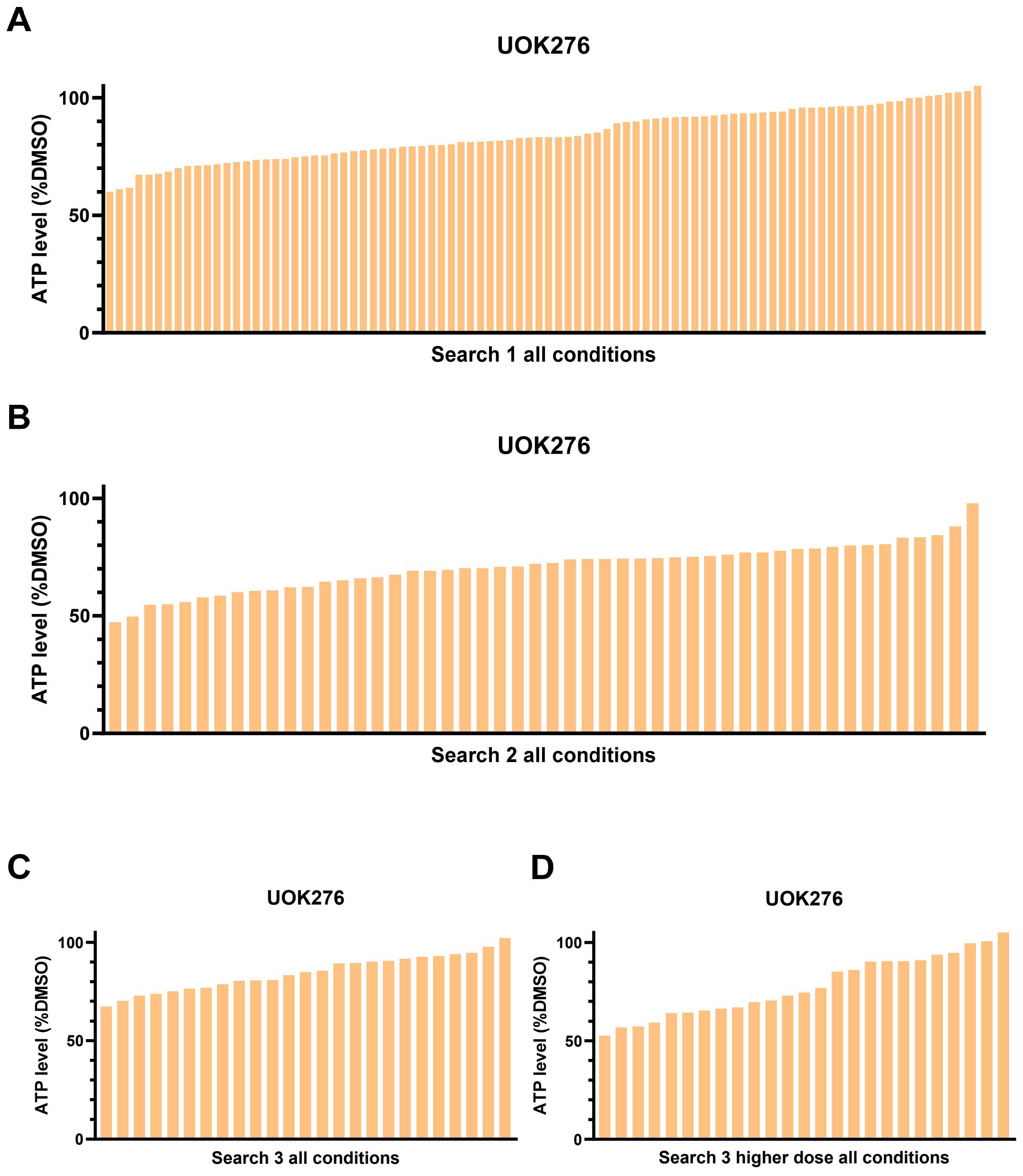

Supplementary Figure S6. Sorted experimental measurements of searches 1-3 of the TGMO-based screen on UOK276 cells. Sorted ATP level averages from lowest to highest of all conditions tested in searches **A**) 1, **B**) 2, and **C,D**) 3 of the TGMO screening on UOK276 cells.

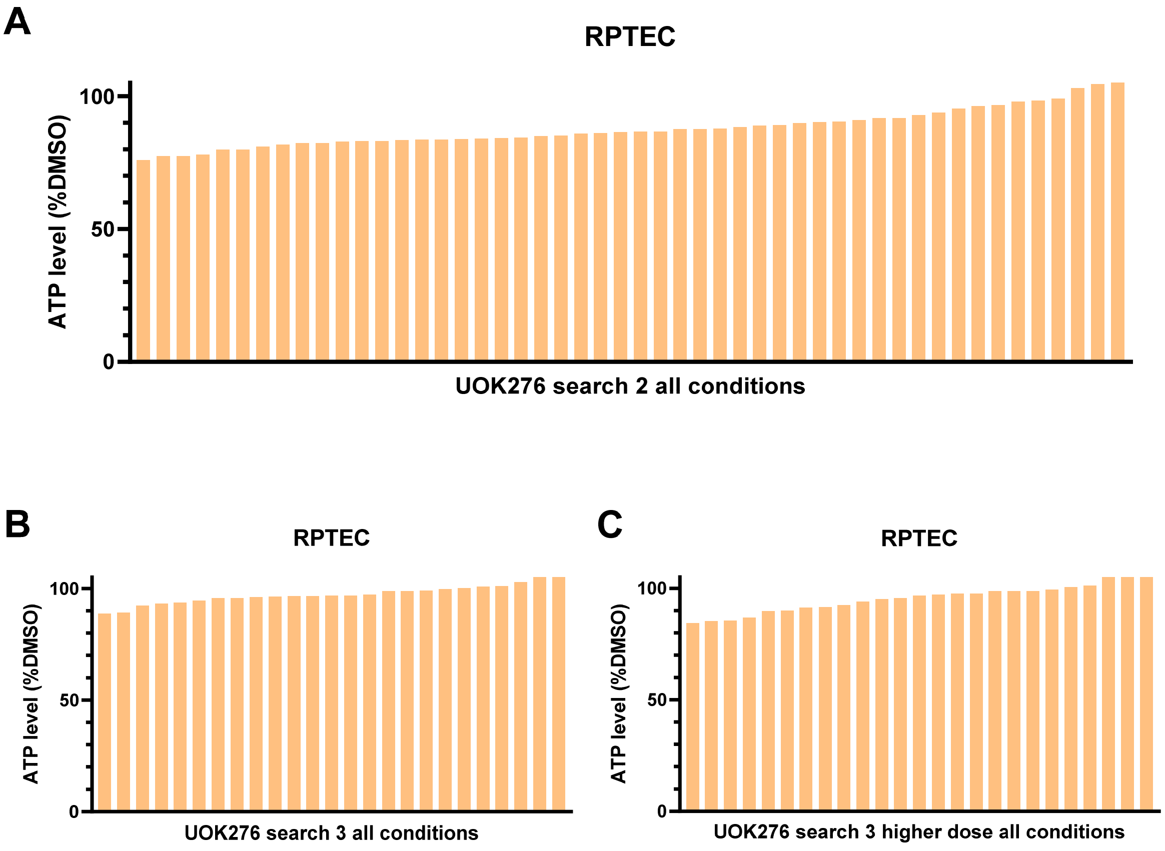

Supplementary Figure S7. Sorted experimental measurements of searches 2 and 3 of the TGMO-based screen in RPTEC cells as part of the UOK276 TGMO-based screening. Sorted ATP level averages from lowest to highest of all conditions tested in RPTEC cells in searches **A**) 2, and **B,C**) 3 of the TGMO screening on UOK276 cells.

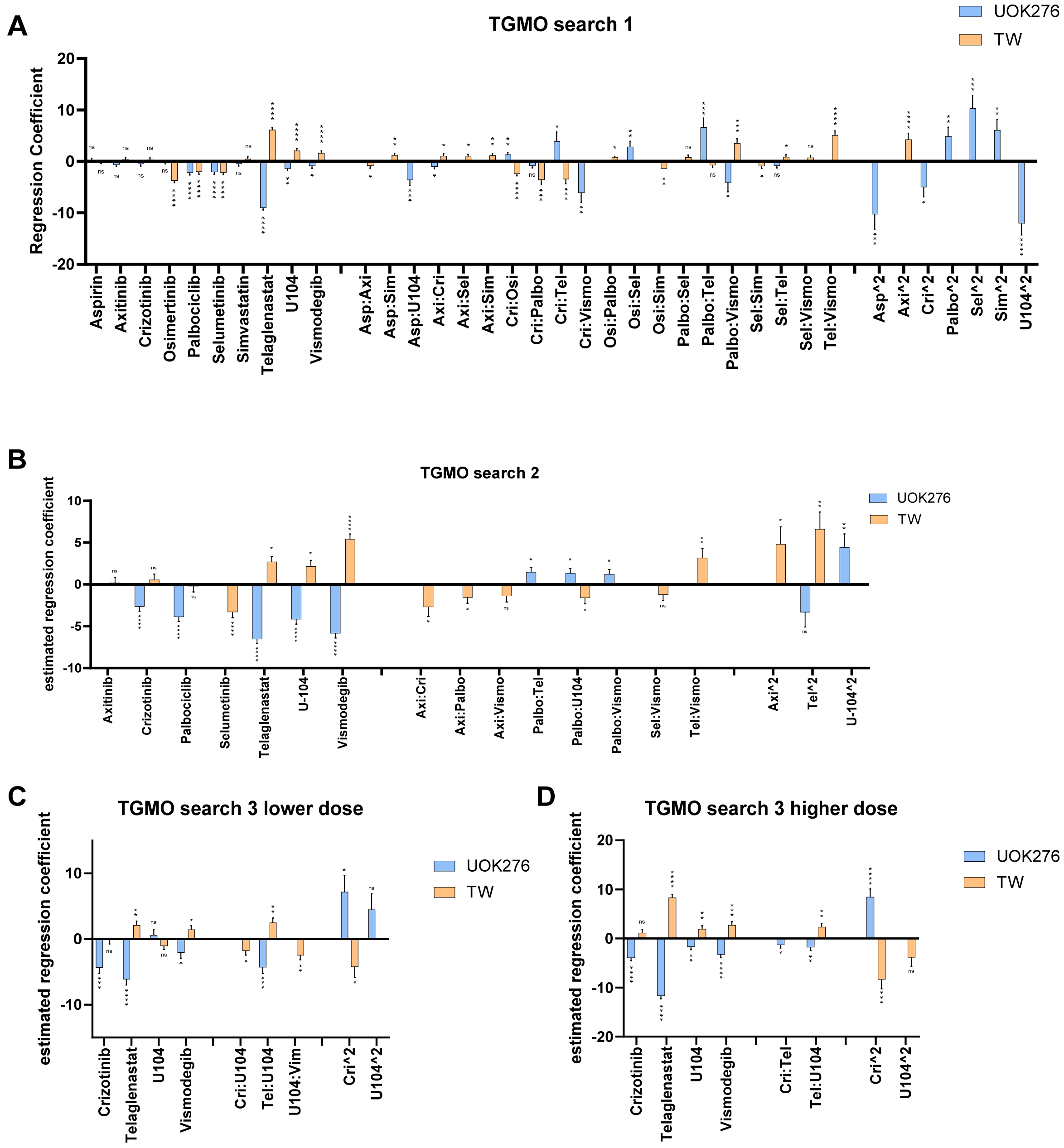

Supplementary Figure S8. Searches 1-3 of the TGMO-based screen in the UOK276 cells and RPTEC cells. Regression coefficients are estimated from a computational model based on experimental data input of the **A**) search 1, **B**) search 2, and **C,D**) search 3. Tables are separated by empty thick into three parts representing (from left to right) 1st order single drug activity, drug-drug interactions, and 2nd order single drug activity. Graphs display the effect on UOK276 cells (blue) and therapeutic window (TW) – calculated as the difference between the experimental effect on healthy RPTEC cells and cancerous UOK276 cells. Error bars and stars respectively correspond to the standard deviation and significance of the estimated regression coefficients. All screenings were performed as N=3 independent experiments. Abbreviations: Aspirin (Asp), axitinib (Axi), crizotinib (Cri), osimertinib (Osi), palbociclib (Palbo), selumetinib (Sel), simvastatin (Sim), telaglenastat (Tel), vismodegib (Vismo).

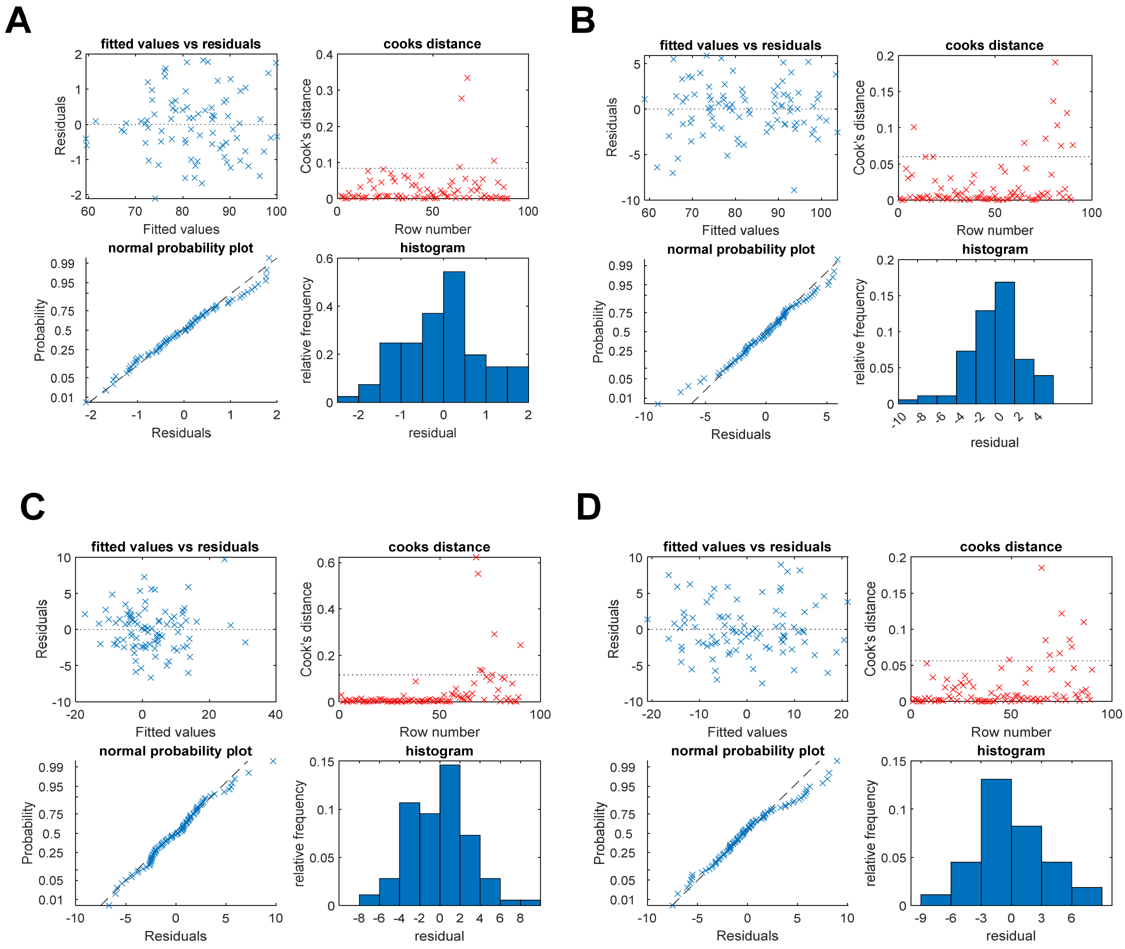

Supplementary Figure S9. Statistical output of the linear regression models to interpret the TGMO-based search in **A**) 786O cells search 1, **B**) UOK276 cells search 1, **C**) TW of the 786O search 1, and **D**) TW of the UOK276 search 1. **A-D**) plots included: Residual analysis plot (top left), Cook's distance plot for outlier detection (top right), Quantile-Quantile plot of probability distribution (bottom left), and histogram of the residual's distribution (bottom right).

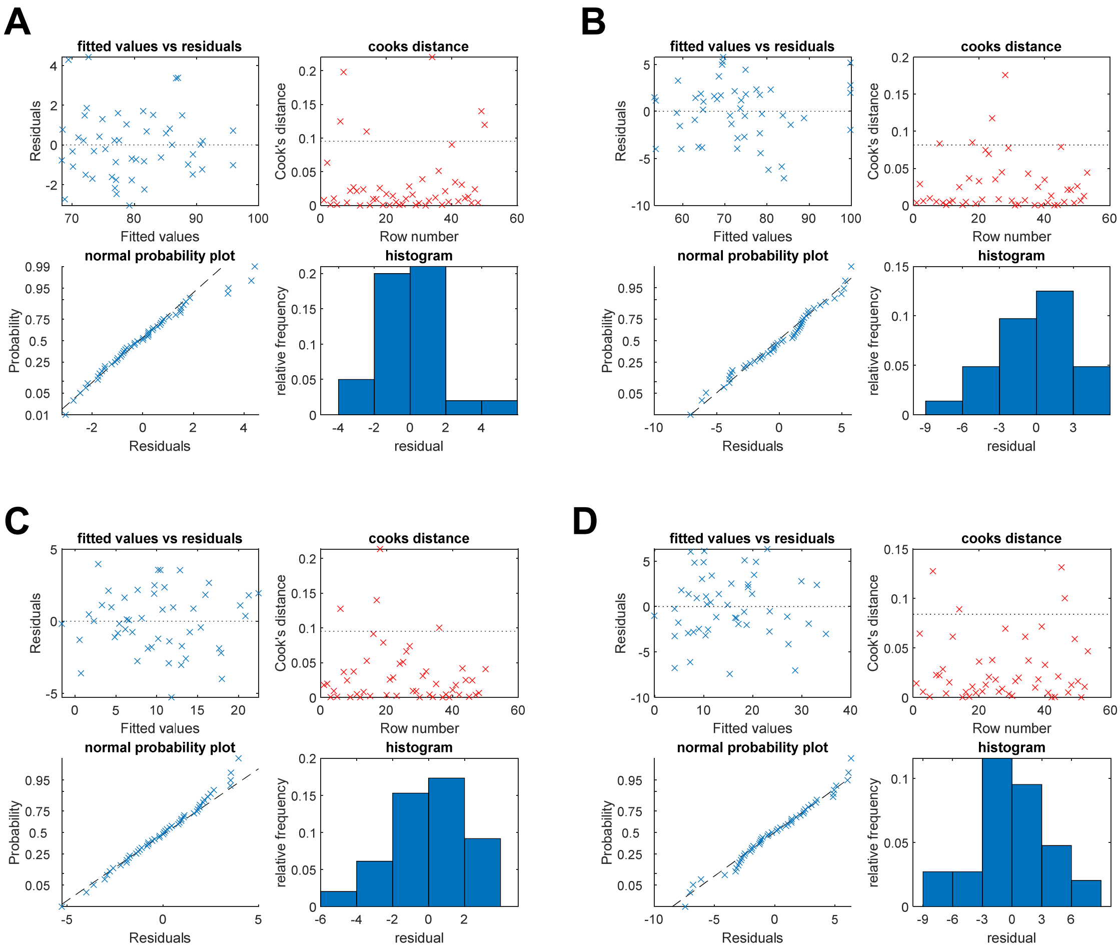

Supplementary Figure S10. Statistical output of the linear regression models to interpret the TGMO-based search in **A**) 786O cells search 2, **B**) UOK276 cells search 2, **C**) TW of the 786O search 2, and **D**) TW of the UOK276 search 2. **A-D**) plots included: Residual analysis plot (top left), Cook's distance plot for outlier detection (top right), Quantile-Quantile plot of probability distribution (bottom left), and histogram of the residual's distribution (bottom right).

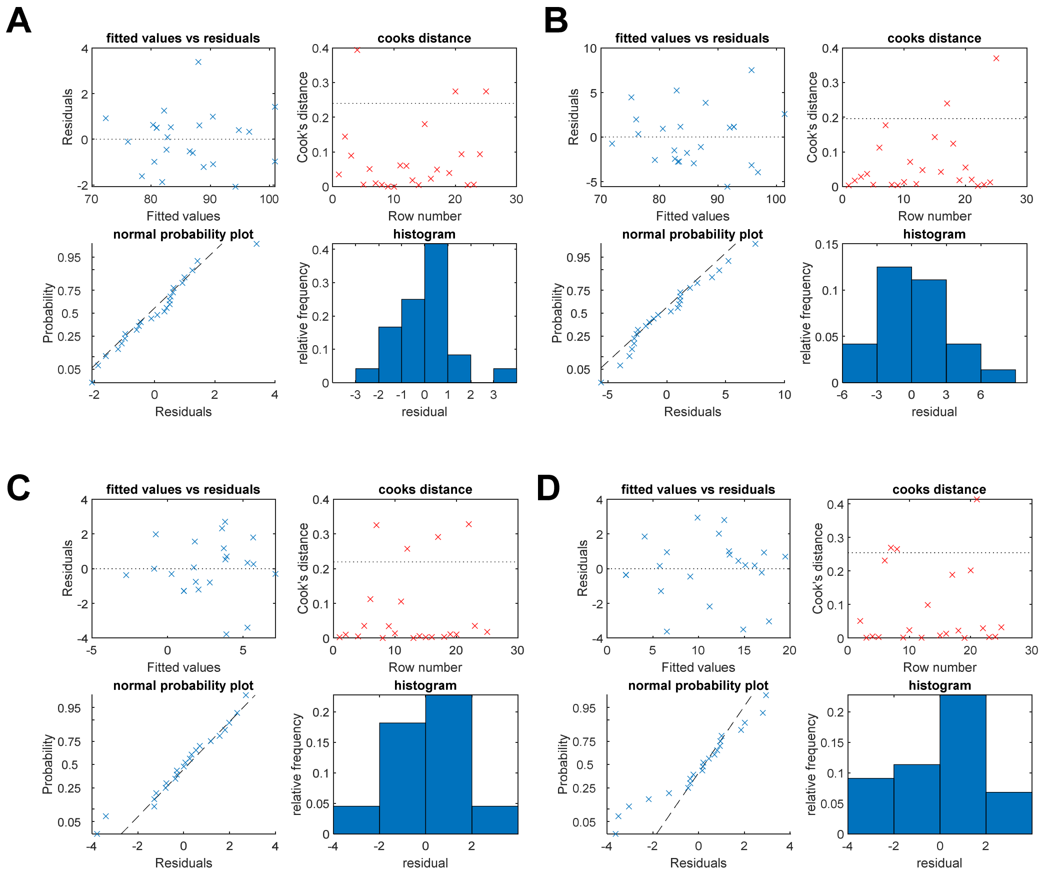

Supplementary Figure S11. Statistical output of the linear regression models to interpret the TGMO-based search in **A**) 786O cells search 3, **B**) UOK276 cells search 3, **C**) TW of the 786O search 3, and **D**) TW of the UOK276 search 3 all from the screen with higher doses of the drugs. **A-D**) plots included: Residual analysis plot (top left), Cook's distance plot for outlier detection (top right), Quantile-Quantile plot of probability distribution (bottom left), and histogram of the residual's distribution (bottom right).

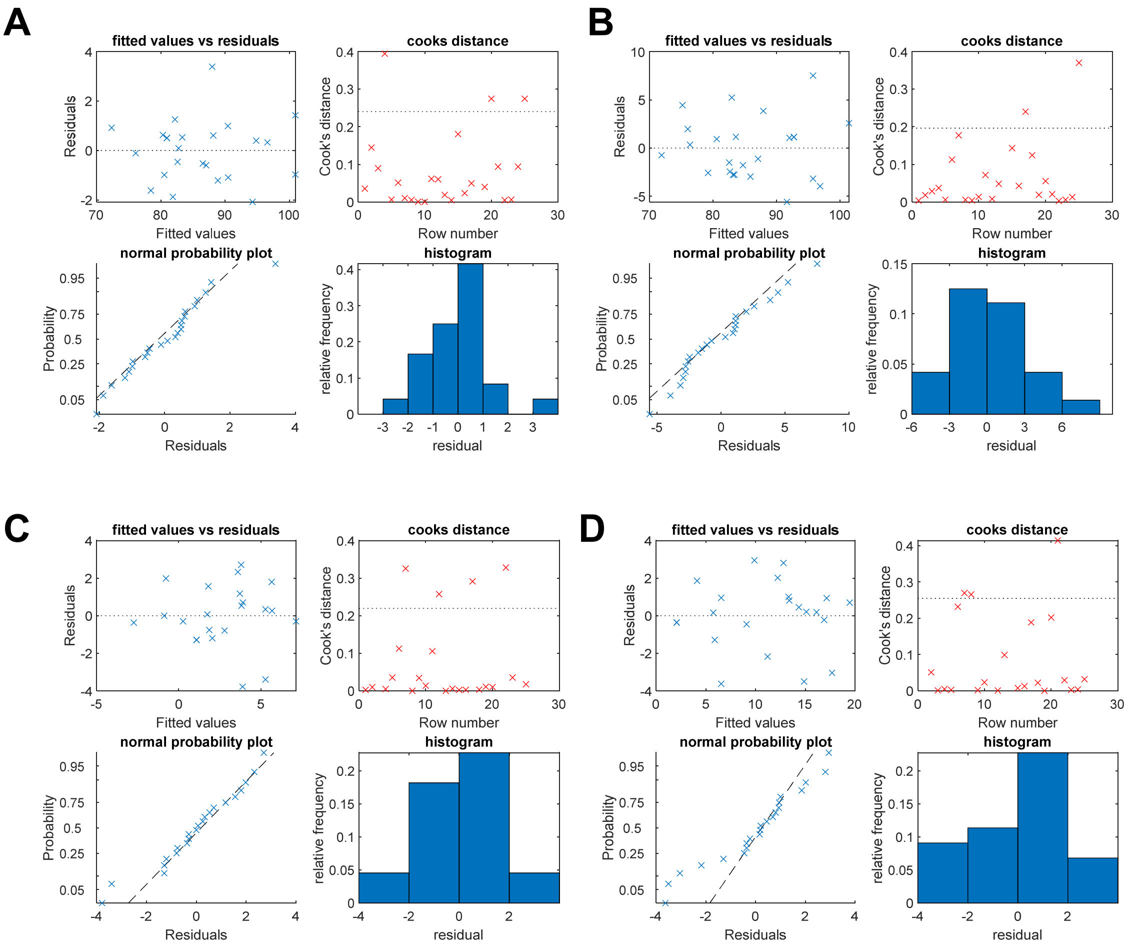

Supplementary Figure S12. Statistical output of the linear regression models to interpret the TGMO-based search in **A**) 786O cells search 3, **B**) UOK cells search 3, **C**) TW of the 786O search 3, and **D**) TW of the UOK276 search 3 all from the screen with original doses of the drugs. **A-D**) plots included: Residual analysis plot (top left), Cook's distance plot for outlier detection (top right), Quantile-Quantile plot of probability distribution (bottom left), and histogram of the residual's distribution (bottom right).

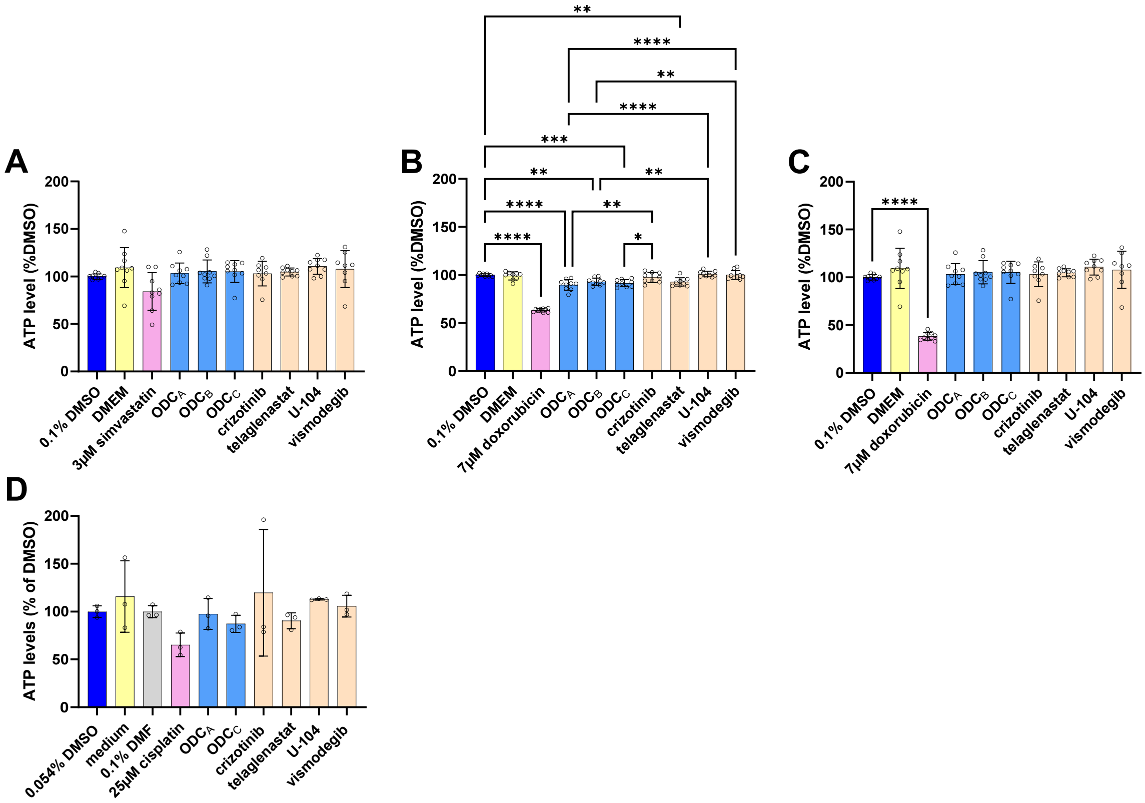

Supplementary Figure S13. Safety profile of the ODCs in non-differentiated or semi-differentiated models. ATP level of **A**) non-differentiated HepaRG cells, **B**) non-differentiated H9c2 cells, **C**) H9c2 cells differentiated with 10 nM of all-trans-retinoic acid for two weeks, and **D**) patient-derived kidney organoids (PHK27). **A-D**) All models were treated with either the ODCs (light blue), corresponding monotherapies (orange), positive (pink), or negative controls (dark blue and yellow). Error bars correspond to the standard deviation of **A-C**) N=3 independent experiments or **D**) N=1 independent experiment. Circles highlight individual technical replicas n=2-3. All results are displayed as a percentage of the DMSO vehicle control (dark blue). **A-C**) Significance was calculated with One-way ANOVA with Šídák's multiple comparisons test and displayed as *p < 0.05, **p < 0.01, ***p < 0.001, and ****p < 0.0001.

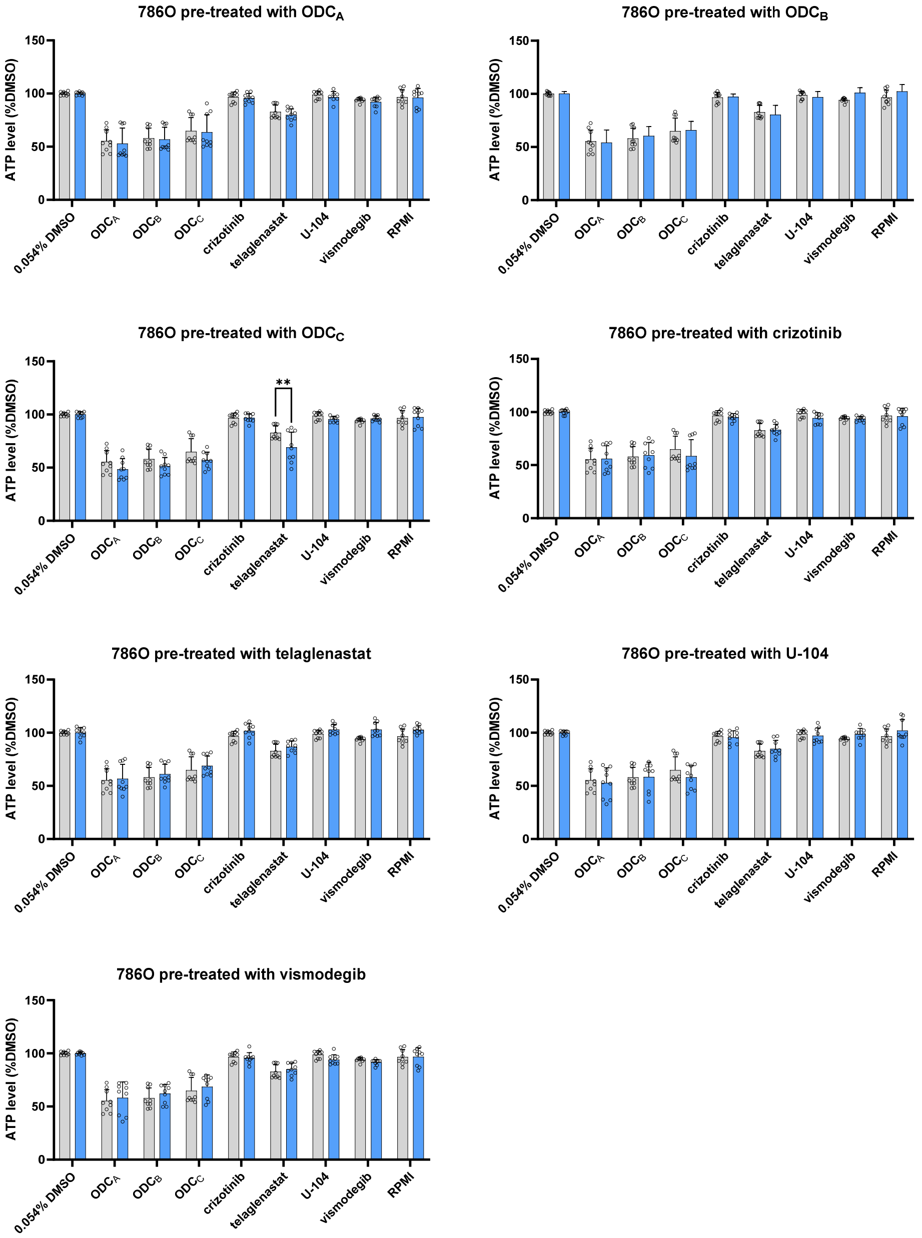

Supplementary Figure S14. Effect of re-treatment on 786O cells. For each graph, cells were treated for 72 hours with one of the nine conditions. After this first treatment, cells were seeded in 96-well plates and retreated with all the different treatment conditions. All graphs are the results of N=3 independent experiments including n=3 technical replicate for each condition. Grey bars show the ATP levels of 786O cells pre-treated with the 0.054% DMSO control then treated with the ODCs, controls, or monotherapies. Displayed data are normalized as a percentage of the corresponding 0.054% DMSO control of the first treatment. Blue bars show ATP levels of 786O cells retreated, also normalized as a percentage of the corresponding 0.054% DMSO control after the second treatment. Significance was calculated with Two-way ANOVA with Tukey's multiple comparisons test comparing the grey and blue bars of each treatment condition. **$p < 0.01$.

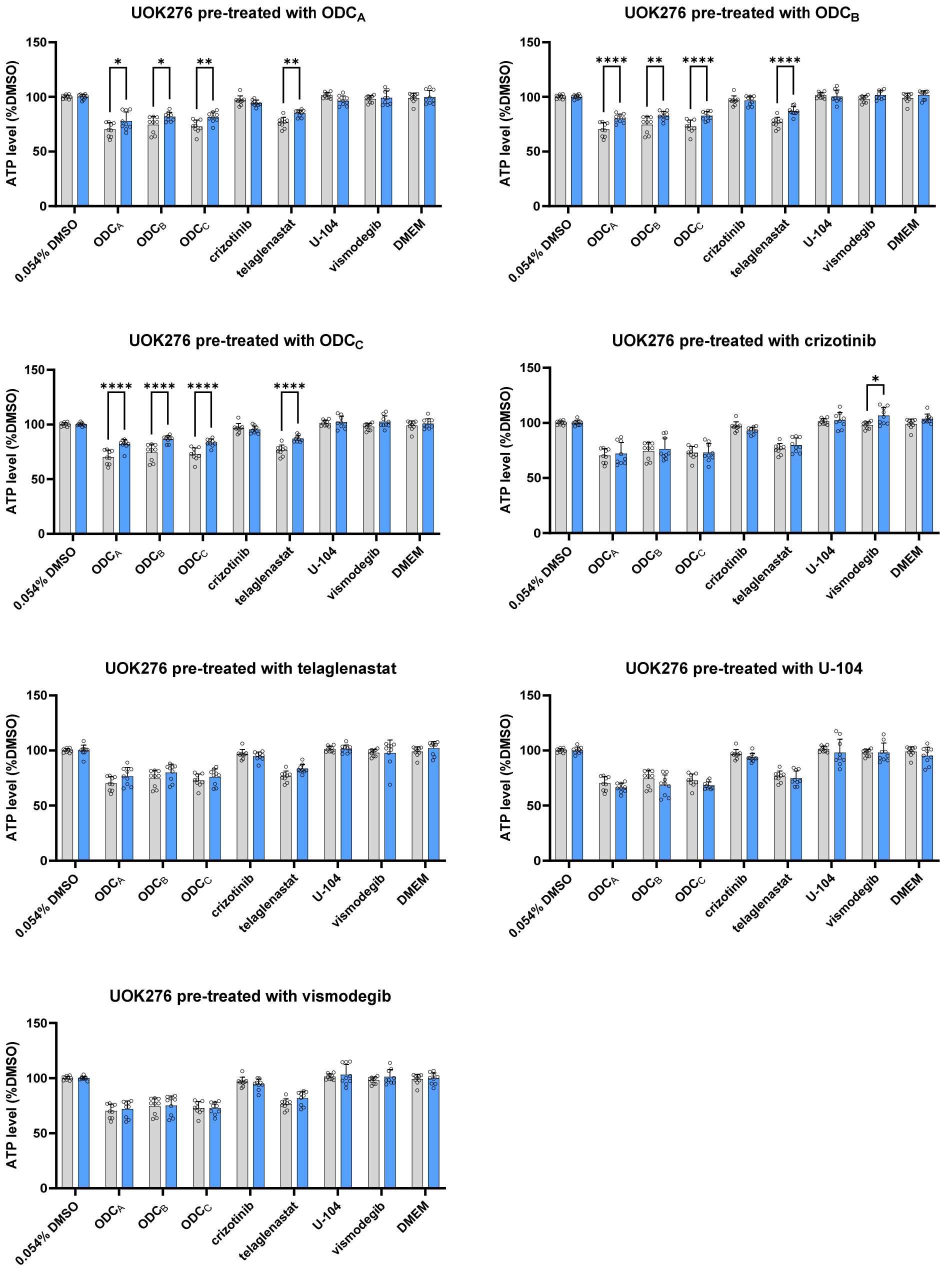

Supplementary Figure S15. Effect of re-treatment on UOK276 cells. For each graph, cells were treated for 72 hours with one of the nine conditions. After this first treatment, cells were seeded in 96-well plates and retreated with all the different treatment conditions. All graphs are the results of N=3 independent experiments including n=3 technical replicate for each condition. Grey bars show the ATP levels of UOK276 cells pre-treated with the 0.054% DMSO control then treated with the ODCs, controls, or monotherapies. Displayed data are normalized as a percentage of the corresponding 0.054% DMSO control of the first treatment. Blue bars show ATP levels of UOK276 cells retreated, also normalized as a percentage of the corresponding 0.054% DMSO control after the second treatment. Significance was calculated with Two-way ANOVA with Tukey's multiple comparisons test comparing the grey and blue bars of each treatment condition. *p < 0.05, **p < 0.01, ****p < 0.001.

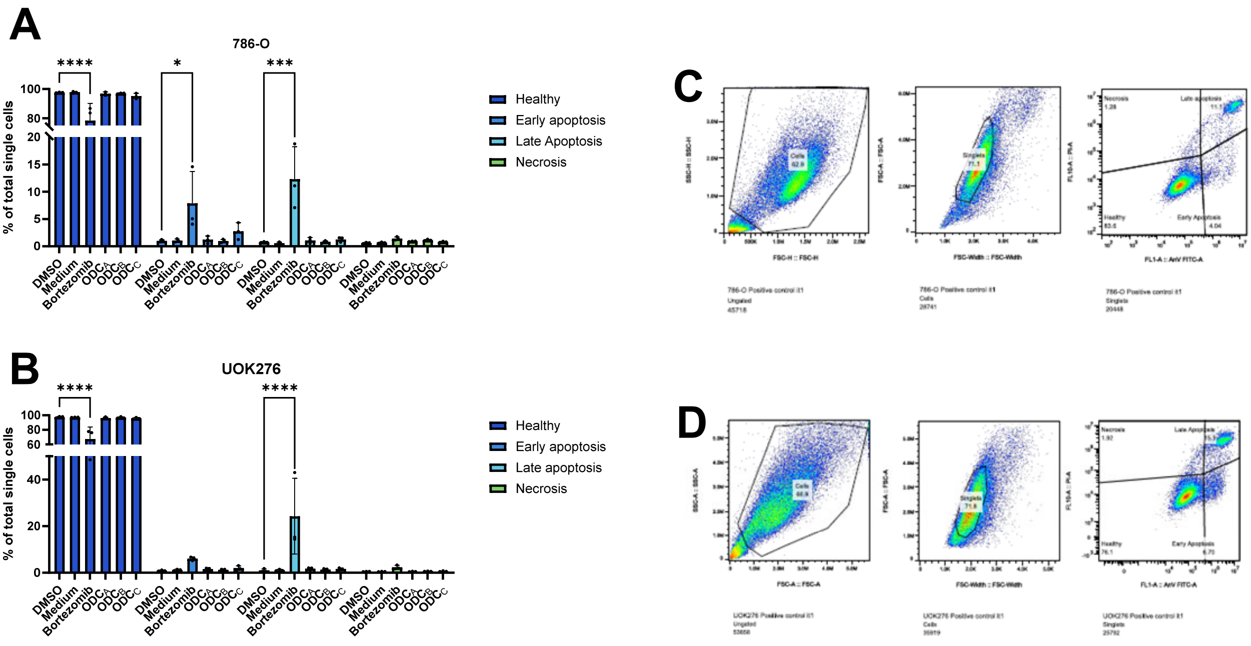

Supplementary Figure S16. Cell death assay analysis on treated 786-O (A) and UOK276 (B) cells. A and B percentage of healthy/early apoptotic/late apoptotic/necrotic cells as determined by AV/PI staining. **A and B** error bars indicate the standard deviation (N=3 independent experiments). Significance was calculated using Two-way ANOVA test and displayed as *p < 0.05, **p < 0.01, ***p < 0.001, and ****p < 0.0001. **C and D** Example of gating strategies used for cell death assay analysis of **(C)** 786-O and **(D)** UOK276 cells. **C and D** Cells are isolated from debris using the size and granularity information brought by the forward and sideward scatter signals (FSC and SSC). Singlets are further isolated based on FSC-A vs FSC-W. FL 10-A PI-A corresponds to the intensity of propidium iodide staining signal, while FL 1-A AnV FITC-A corresponds to the intensity of annexin V staining signal.

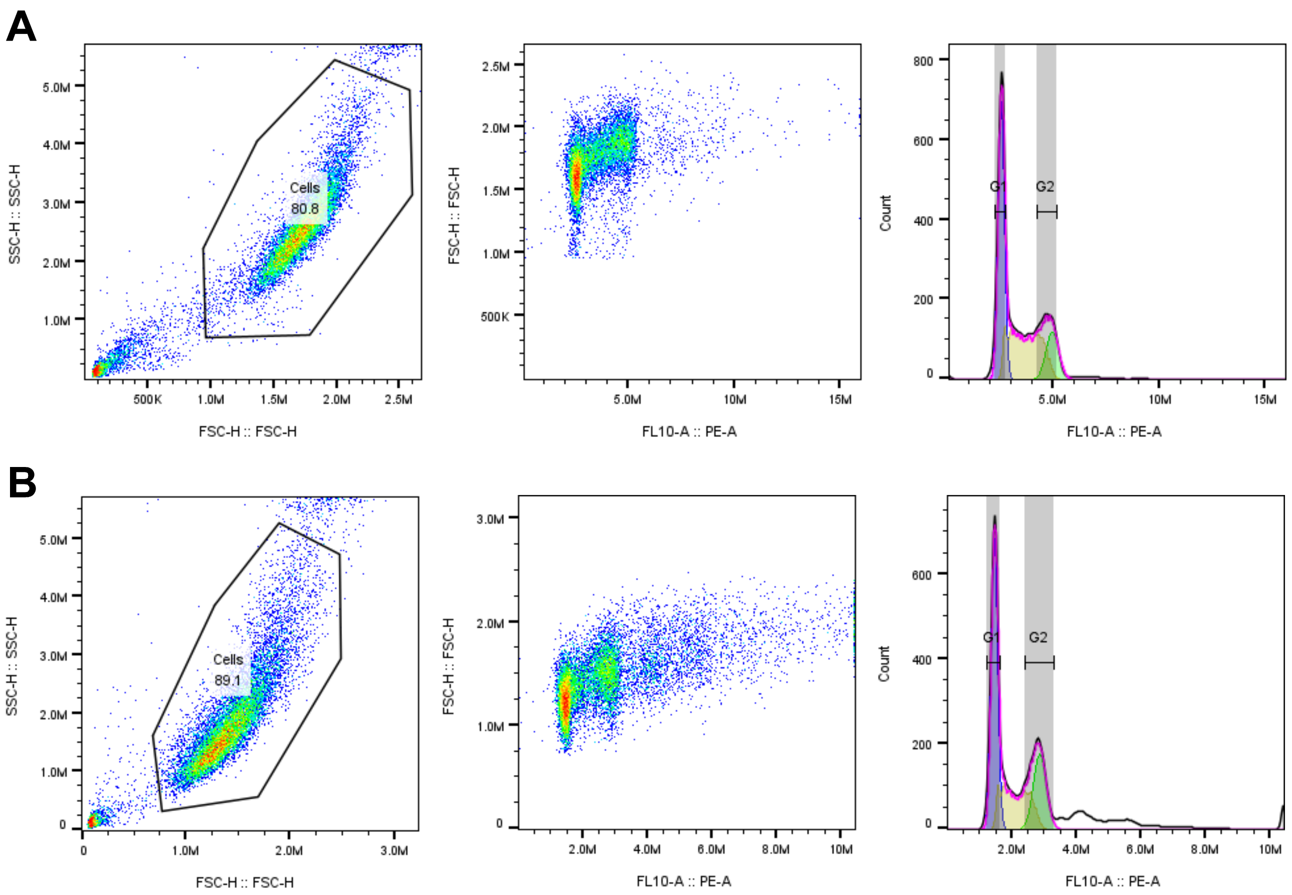

Supplementary Figure S17. Gating procedure used for the cell cycle analysis. Example of the gating used for the cell cycle analysis of **A**) 786O cells and **B**) UOK276 cells. **A** and **B**) Cells are isolated from debris using the size and granularity information brought by the forward and sideward scatter signals (FSC and SSC). FL10-A PE-A corresponds to the intensity of the propidium iodide staining signal. Cell cycle phases were fitted with the Watson (pragmatic) model from the FlowJo 10.10.0 embedded cell cycle tool.

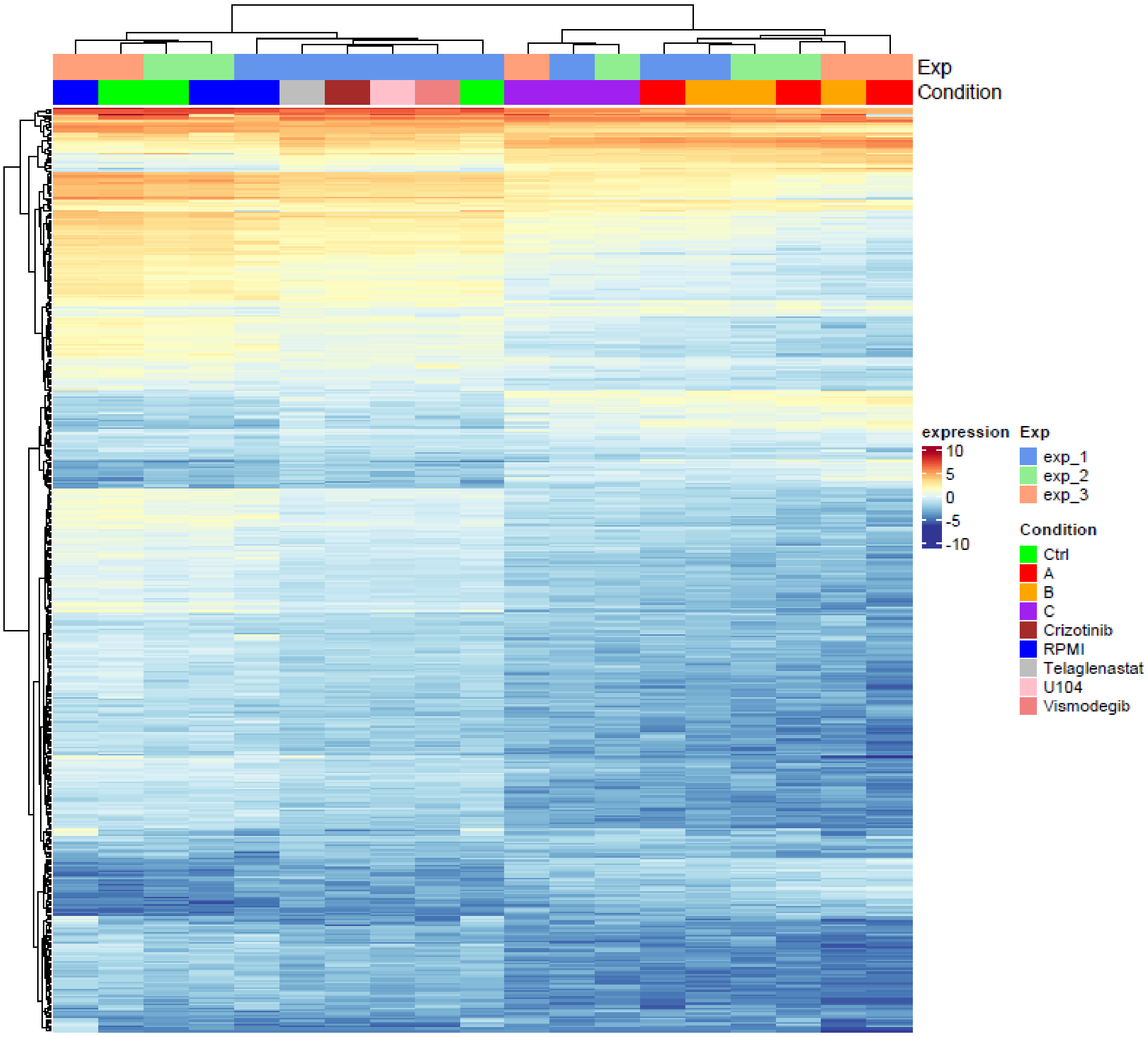

Supplementary Figure S18. Heatmap of the top 500 most variable genes across samples in treated 786O cells corrected for batch effect. Color code for change in expression (expression), experimental batch (Exp), and treatment conditions (Condition) can be found on the right side of the heatmap.

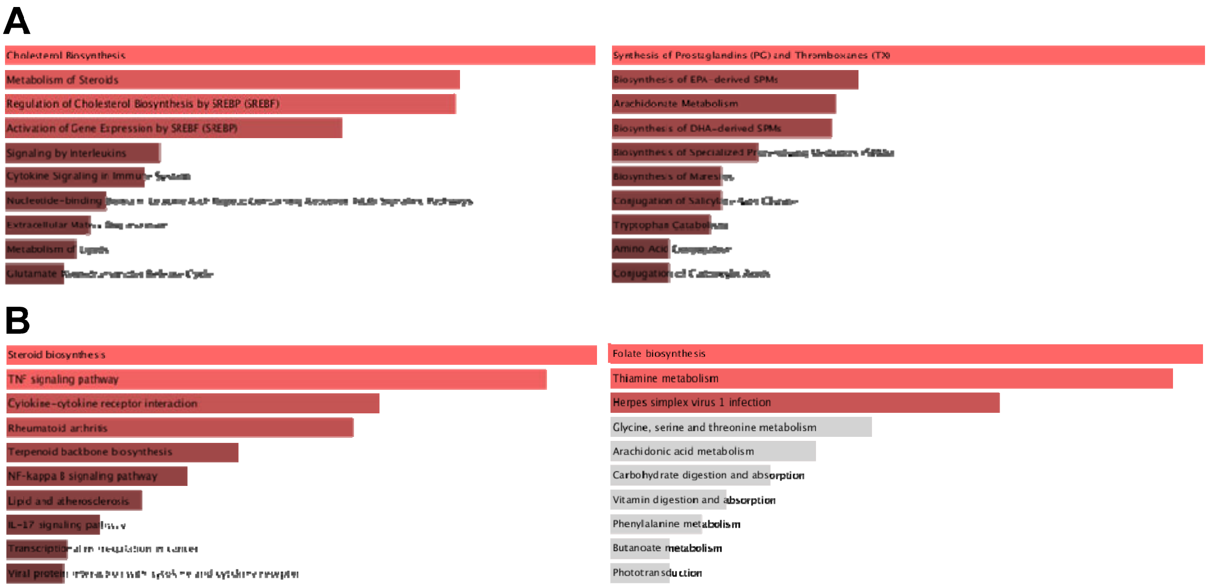

Supplementary Figure S19. Pathways linked to the changes observed in the RNA sequencing data of 786O cells treated with ODC$_A$. Significantly upregulated genes (left) and downregulated genes (right) were entered separately into the Enrichr web-based analysis tool to generate this figure **A**) from Reactome Pathways 2024, and **B**) from KEGG 2021 Human. All displayed pathways in the bar graphs are ranked based on their p-value in the enrichment analysis. Grey bars are not significant (p-value > 0.05).

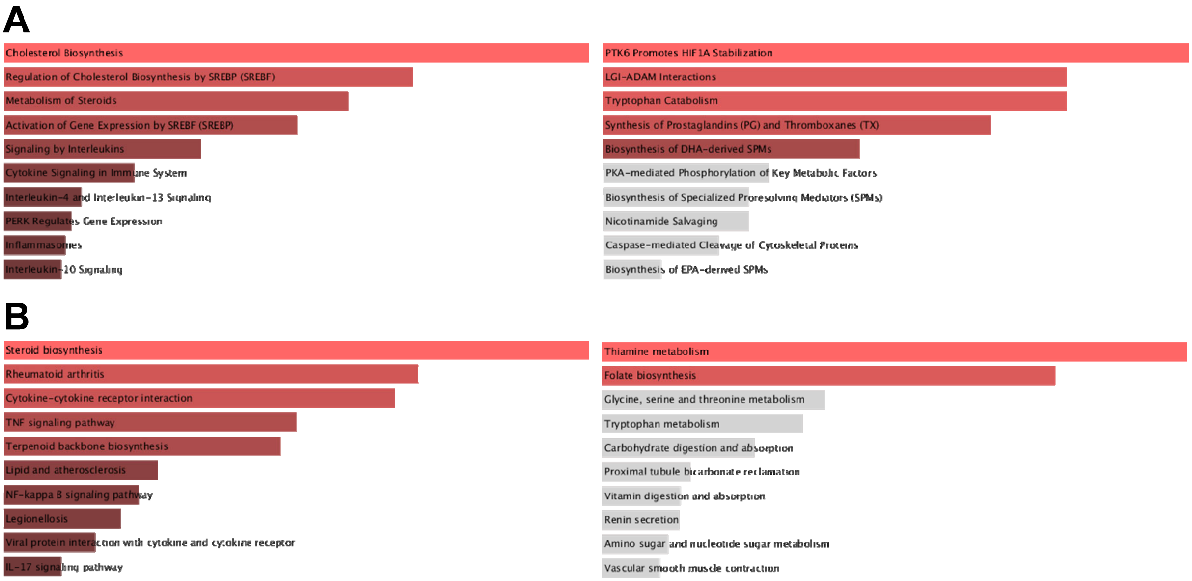

Supplementary Figure S20: Pathways linked to the changes observed in the RNA sequencing data of 786O cells treated with ODC$_B$. Significantly upregulated genes (left) and downregulated genes (right) were entered separately into the Enrichr web-based analysis tool to generate this figure **A**) from Reactome Pathways 2024, and **B**) from KEGG 2021 Human. All displayed pathways in the bar graphs are ranked based on their p-value in the enrichment analysis. Grey bars are not significant (p-value > 0.05).

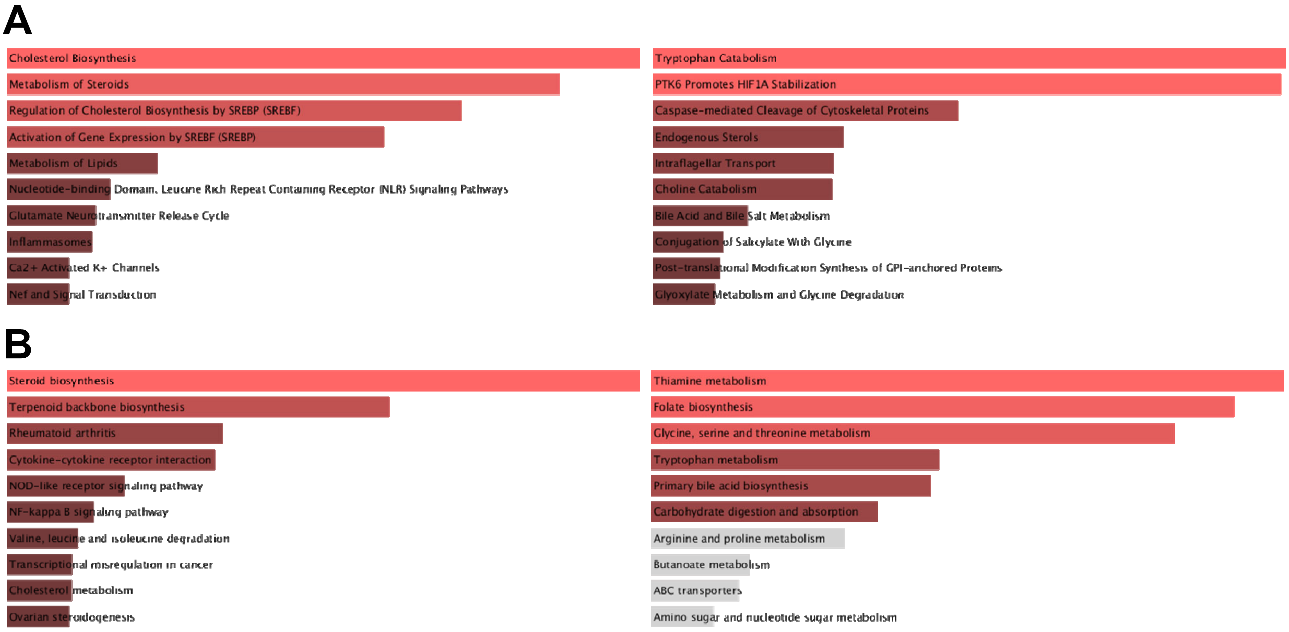

Supplementary Figure S21. Pathways linked to the changes observed in the RNA sequencing data of 786O cells treated with ODC$_C$. Significantly upregulated genes (left) and downregulated genes (right) were entered separately into the Enrichr web-based analysis tool to generate this figure **A**) from Reactome Pathways 2024, and **B**) from KEGG 2021 Human. All displayed pathways in the bar graphs are ranked based on their p-value in the enrichment analysis. Grey bars are not significant (p-value > 0.05).

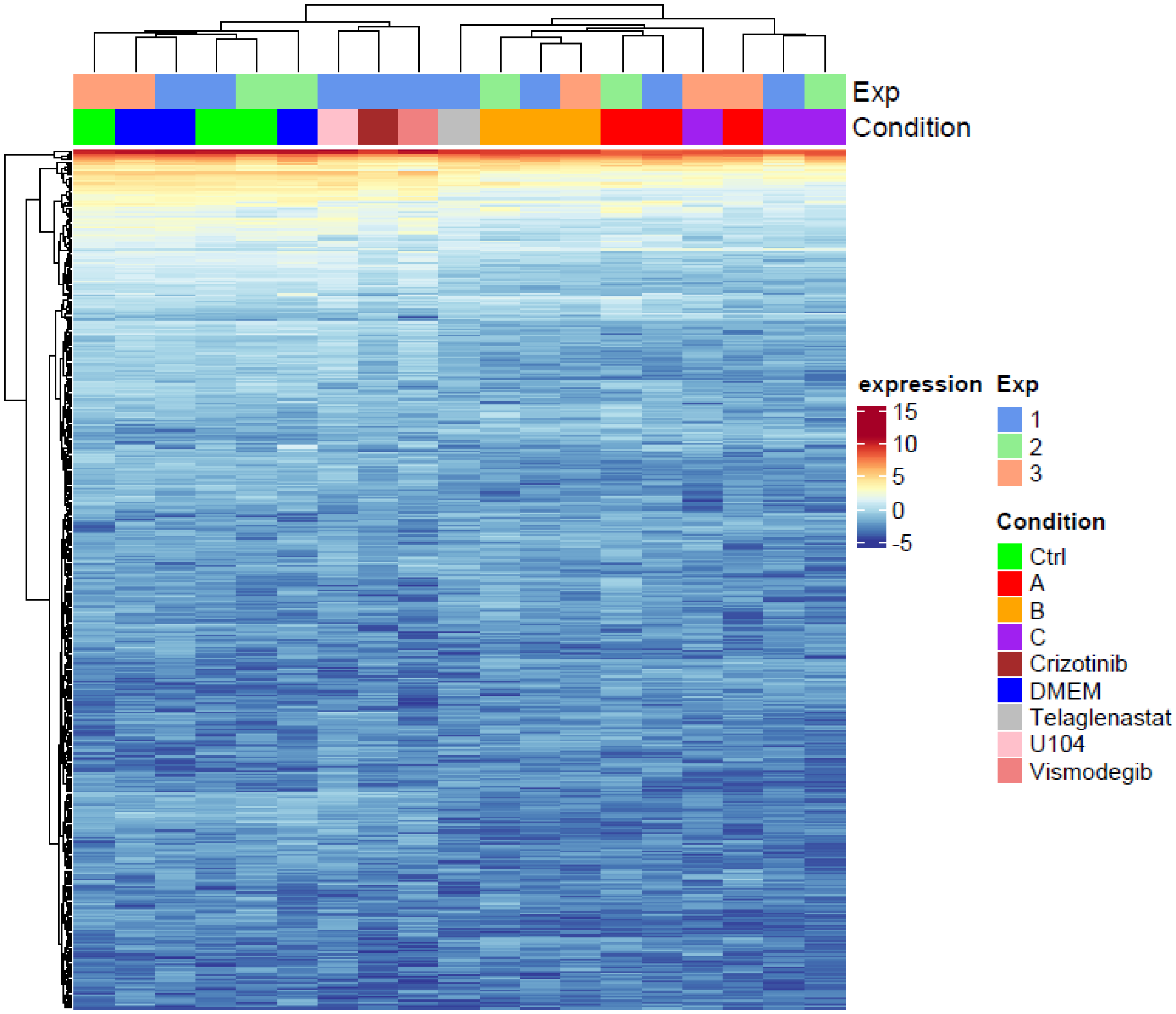

Supplementary Figure S22. Heatmap of the top 500 most variable genes across samples in treated UOK276 cells corrected for batch effect. Color code for change in expression (expression), experimental batch (Exp), and treatment conditions (Condition) can be found on the right side of the heatmap.

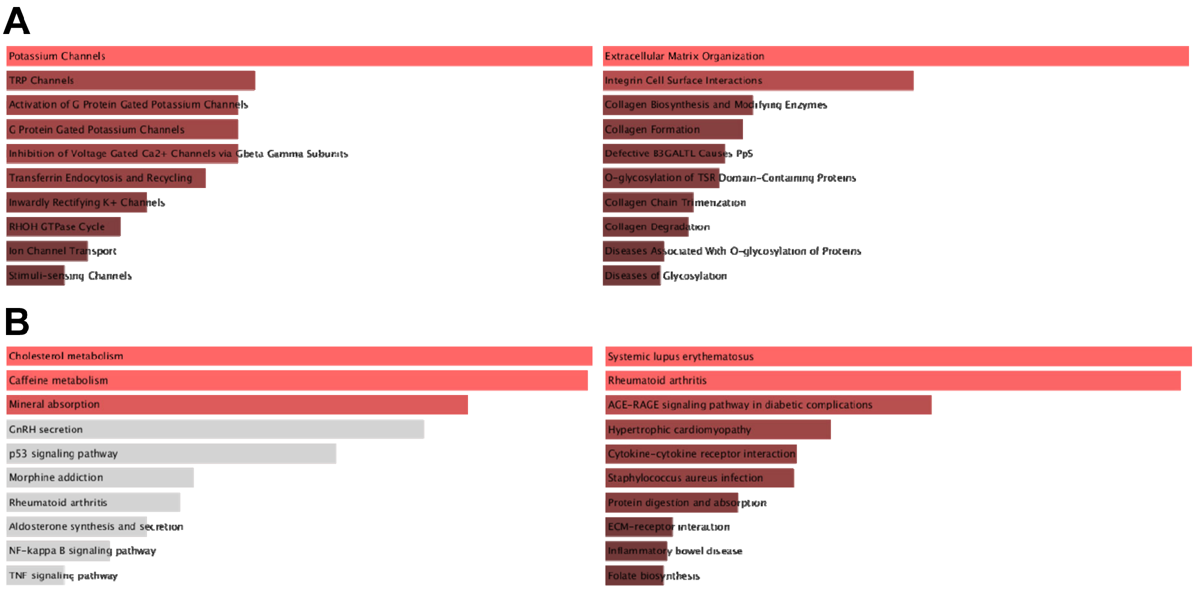

Supplementary Figure S23. Pathways linked to the changes observed in the RNA sequencing data of UOK276 cells treated with ODC$_A$. Significantly upregulated genes (left) and downregulated genes (right) were entered separately into the Enrichr web-based analysis tool to generate figure **A**) from Reactome Pathways 2024, and **B**) from KEGG 2021 Human. All displayed pathways in the bar graphs are ranked based on their p-value in the enrichment analysis. Grey bars are not significant (p-value > 0.05).

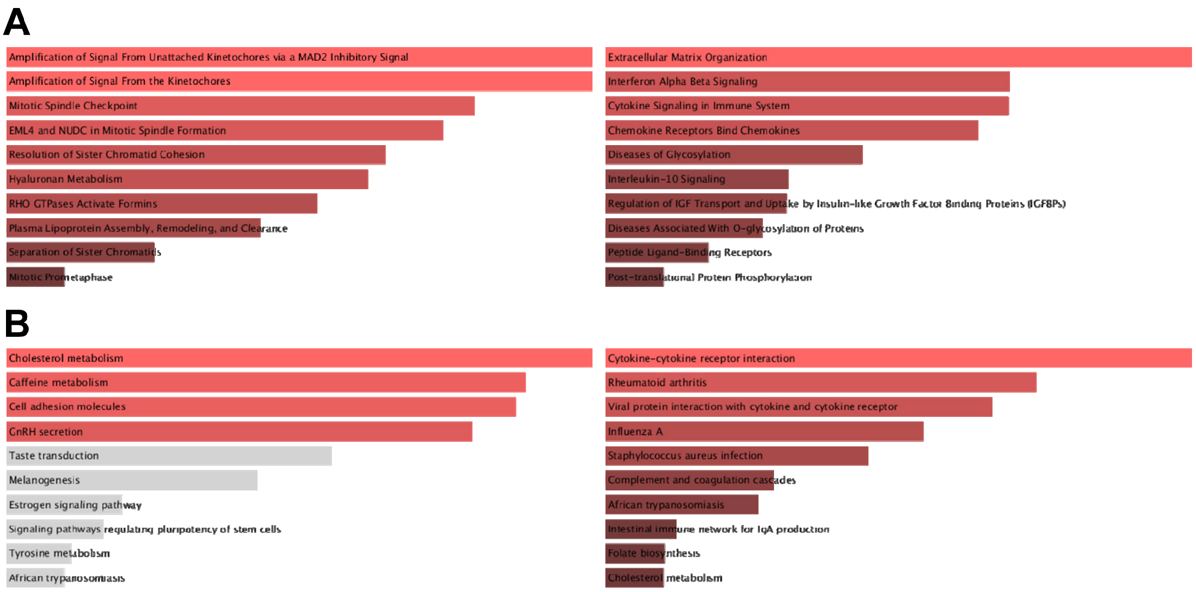

Supplementary Figure S24. Pathways linked to the changes observed in the RNA sequencing data of UOK276 cells treated with ODC$_B$. Significantly upregulated genes (left) and downregulated genes (right) were entered separately into the Enrichr web-based analysis tool to generate this figure **A**) from Reactome Pathways 2024, and **B**) from KEGG 2021 Human. All displayed pathways in the bar graphs are ranked based on their p-value in the enrichment analysis. Grey bars are not significant (p-value > 0.05).

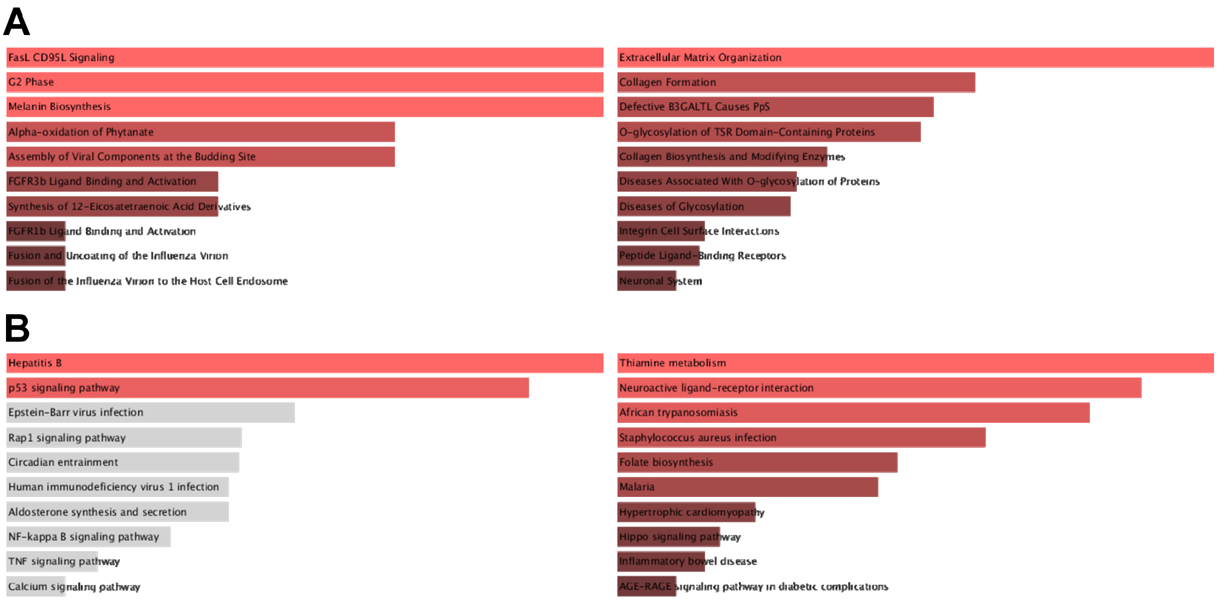

Supplementary Figure S25. Pathways linked to the changes observed in the RNA sequencing data of UOK276 cells treated with ODC$_C$. Significantly upregulated genes (left) and downregulated genes (right) were entered separately into the Enrichr web-based analysis tool to generate this figure **A**) from Reactome Pathways 2024, and **B**) from KEGG 2021 Human. All displayed pathways in the bar graphs are ranked based on their p-value in the enrichment analysis. Grey bars are not significant (p-value > 0.05).

Supplementary Table S1 Pathways linked to the changes observed in the RNA sequencing data of 786O cells treated with ODC$_A$. Significantly upregulated genes were entered into the Enrichr web-based analysis tool to generate this gene enrichment table from Reactome Pathways 2024. Only the first ten entries ranked by p-value are displayed.

TERM	P-VALUE	ADJUSTED P-VALUE	ODDS RATIO
CHOLESTEROL BIOSYNTHESIS	1.29E-13	1.64E-10	25.452
METABOLISM OF STEROIDS	4.13E-11	2.11E-08	4.937
REGULATION OF CHOLESTEROL BIOSYNTHESIS BY SREBP (SREBF)	4.96E-11	2.11E-08	9.663
ACTIVATION OF GENE EXPRESSION BY SREBF (SREBP)	5.84E-09	1.86E-06	9.810
SIGNALING BY INTERLEUKINS	1.25E-05	0.003	2.161
CYTOKINE SIGNALING IN IMMUNE SYSTEM	2.36E-05	0.005	1.827
NUCLEOTIDE-BINDING DOMAIN, LEUCINE RICH REPEAT CONTAINING RECEPTOR (NLR) SIGNALING PATHWAYS	1.24E-04	0.023	4.452
EXTRACELLULAR MATRIX ORGANIZATION	2.39E-04	0.038	2.182
METABOLISM OF LIPIDS	4.21E-04	0.060	1.671
GLUTAMATE NEUROTRANSMITTER RELEASE CYCLE	7.28E-04	0.078	6.720

Supplementary Table S2 Pathways linked to the changes observed in the RNA sequencing data of 786O cells treated with ODC$_A$. Significantly downregulated genes were entered into the Enrichr web-based analysis tool to generate this gene enrichment table from Reactome Pathways 2024. Only the first ten entries ranked by p-value are displayed.

TERM	P-VALUE	ADJUSTED P-VALUE	ODDS RATIO
SYNTHESIS OF PROSTAGLANDINS (PG) AND THROMBOXANES (TX)	0.001	1.000	6.929
BIOSYNTHESIS OF EPA-DERIVED SPMS	0.011	1.000	10.380
ARACHIDONATE METABOLISM	0.013	1.000	2.384
BIOSYNTHESIS OF DHA-DERIVED SPMS	0.013	1.000	4.328
BIOSYNTHESIS OF SPECIALIZED PRORESOLVING MEDIATORS (SPMS)	0.021	1.000	3.709
BIOSYNTHESIS OF MARESINS	0.027	1.000	6.227
CONJUGATION OF SALICYLATE WITH GLYCINE	0.027	1.000	6.227
TRYPTOPHAN CATABOLISM	0.029	1.000	4.153
AMINO ACID CONJUGATION	0.038	1.000	5.189
CONJUGATION OF CARBOXYLIC ACIDS	0.038	1.000	5.189

Supplementary Table S3 Pathways linked to the changes observed in the RNA sequencing data of 786O cells treated with ODC_B. Significantly upregulated genes were entered into the Enrichr web-based analysis tool to generate this gene enrichment table from Reactome Pathways 2024. Only the first ten entries ranked by p-value are displayed.

TERM	P-VALUE	ADJUSTED P-VALUE	ODDS RATIO
CHOLESTEROL BIOSYNTHESIS	1.65E-14	1.96E-11	29.573
REGULATION OF CHOLESTEROL BIOSYNTHESIS BY SREBP (SREBF)	5.32E-11	3.17E-08	10.323
METABOLISM OF STEROIDS	1.03E-09	4.11E-07	4.804
ACTIVATION OF GENE EXPRESSION BY SREBF (SREBP)	1.06E-08	3.15E-06	10.217
SIGNALING BY INTERLEUKINS	8.58E-07	2.05E-04	2.455
CYTOKINE SIGNALING IN IMMUNE SYSTEM	1.84E-05	0.004	1.905
INTERLEUKIN-4 AND INTERLEUKIN-13 SIGNALING	2.09E-04	0.036	3.360
PERK REGULATES GENE EXPRESSION	3.31E-04	0.049	6.303
INFLAMMASOMES	4.37E-04	0.057	7.386
INTERLEUKIN-10 SIGNALING	5.25E-04	0.057	4.931

Supplementary Table S4 Pathways linked to the changes observed in the RNA sequencing data of 786O cells treated with ODC_B. Significantly downregulated genes were entered into the Enrichr web-based analysis tool to generate this gene enrichment table from Reactome Pathways 2024. Only the first ten entries ranked by p-value are displayed.

TERM	P-VALUE	ADJUSTED P-VALUE	ODDS RATIO
PTK6 PROMOTES HIF1A STABILIZATION	0.013	1.000	8.918
LGI-ADAM INTERACTIONS	0.019	1.000	4.758
TRYPTOPHAN CATABOLISM	0.019	1.000	4.758
SYNTHESIS OF PROSTAGLANDINS (PG) AND THROMBOXANES (TX)	0.025	1.000	4.325
BIOSYNTHESIS OF DHA-DERIVED SPMS	0.038	1.000	3.659
PKA-MEDIATED PHOSPHORYLATION OF KEY METABOLIC FACTORS	0.052	1.000	7.922
BIOSYNTHESIS OF SPECIALIZED PRORESOLVING MEDIATORS (SPMS)	0.055	1.000	3.171
NICOTINAMIDE SALVAGING	0.055	1.000	3.171
CASPASE-MEDIATED CLEAVAGE OF CYTOSKELETAL PROTEINS	0.061	1.000	3.962
BIOSYNTHESIS OF EPA-DERIVED SPMS	0.073	1.000	5.941

Supplementary Table S5 Pathways linked to the changes observed in the RNA sequencing data of 786O cells treated with ODC$_C$. Significantly upregulated genes were entered into the Enrichr web-based analysis tool to generate this gene enrichment table from Reactome Pathways 2024. Only the first ten entries ranked by p-value are displayed.

TERM	P-VALUE	ADJUSTED P-VALUE	ODDS RATIO
CHOLESTEROL BIOSYNTHESIS	5.10E-18	4.27E-15	52.673
METABOLISM OF STEROIDS	5.38E-16	2.26E-13	9.071
REGULATION OF CHOLESTEROL BIOSYNTHESIS BY SREBP (SREBF)	1.53E-13	4.27E-11	16.868
ACTIVATION OF GENE EXPRESSION BY SREBF (SREBP)	1.34E-11	2.81E-09	18.165
METABOLISM OF LIPIDS	6.37E-06	0.001	2.323
NUCLEOTIDE-BINDING DOMAIN, LEUCINE RICH REPEAT CONTAINING RECEPTOR (NLR) SIGNALING PATHWAYS	9.73E-05	0.014	6.253
GLUTAMATE NEUROTRANSMITTER RELEASE CYCLE	2.23E-04	0.027	10.851
INFLAMMASOMES	2.73E-04	0.029	10.308
CA2+ ACTIVATED K+ CHANNELS	0.001	0.086	20.543
NEF AND SIGNAL TRANSDUCTION	0.001	0.086	20.543

Supplementary Table S6 Pathways linked to the changes observed in the RNA sequencing data of 786O cells treated with ODC$_C$. Significantly downregulated genes were entered into the Enrichr web-based analysis tool to generate this gene enrichment table from Reactome Pathways 2024. Only the first ten entries ranked by p-value are displayed.

TERM	P-VALUE	ADJUSTED P-VALUE	ODDS RATIO
TRYPTOPHAN CATABOLISM	0.002	0.973	9.106
PTK6 PROMOTES HIF1A STABILIZATION	0.002	0.973	17.058
CASPASE-MEDIATED CLEAVAGE OF CYTOSKELETAL PROTEINS	0.012	1.000	7.580
ENDOGENOUS STEROLS	0.023	1.000	4.136
INTRAFLAGELLAR TRANSPORT	0.024	1.000	2.907
CHOLINE CATABOLISM	0.024	1.000	11.359
BILE ACID AND BILE SALT METABOLISM	0.037	1.000	2.918
CONJUGATION OF SALICYLATE WITH GLYCINE	0.042	1.000	7.572
POST-TRANSLATIONAL MODIFICATION SYNTHESIS OF GPI-ANCHORED PROTEINS	0.043	1.000	2.144
GLYOXYLATE METABOLISM AND GLYCINE DEGRADATION	0.044	1.000	4.262

Supplementary Table S7 Pathways linked to the changes observed in the RNA sequencing data of UOK276 cells treated with ODC$_A$. Significantly upregulated genes were entered into the Enrichr web-based analysis tool to generate this gene enrichment table from Reactome Pathways 2024. Only the first ten entries ranked by p-value are displayed.

TERM	P-VALUE	ADJUSTED P-VALUE	ODDS RATIO
POTASSIUM CHANNELS	0.003	0.392	7.202
TRP CHANNELS	0.011	0.392	13.519
ACTIVATION OF G PROTEIN GATED POTASSIUM CHANNELS	0.012	0.392	13.017
G PROTEIN GATED POTASSIUM CHANNELS	0.012	0.392	13.017
INHIBITION OF VOLTAGE GATED CA2+ CHANNELS VIA GBETA GAMMA SUBUNITS	0.012	0.392	13.017
TRANSFERRIN ENDOCYTOSIS AND RECYCLING	0.014	0.392	12.118
INWARDLY RECTIFYING K+ CHANNELS	0.017	0.392	10.647
RHOH GTPASE CYCLE	0.019	0.392	10.038
ION CHANNEL TRANSPORT	0.022	0.392	3.945
STIMULI-SENSING CHANNELS	0.024	0.392	5.095

Supplementary Table S8 Pathways linked to the changes observed in the RNA sequencing data of UOK276 cells treated with ODC$_A$. Significantly downregulated genes were entered into the Enrichr web-based analysis tool to generate this gene enrichment table from Reactome Pathways 2024. Only the first ten entries ranked by p-value are displayed.

TERM	P-VALUE	ADJUSTED P-VALUE	ODDS RATIO
EXTRACELLULAR MATRIX ORGANIZATION	1.93E-10	1.38E-07	5.303
INTEGRIN CELL SURFACE INTERACTIONS	3.30E-07	1.18E-04	8.407
COLLAGEN BIOSYNTHESIS AND MODIFYING ENZYMES	2.48E-05	0.006	7.609
COLLAGEN FORMATION	3.29E-05	0.006	6.246
DEFECTIVE B3GALTL CAUSES PPS	5.28E-05	0.007	10.476
O-GLYCOSYLATION OF TSR DOMAIN-CONTAINING PROTEINS	6.15E-05	0.007	10.158
COLLAGEN CHAIN TRIMERIZATION	1.23E-04	0.013	8.819
COLLAGEN DEGRADATION	1.41E-04	0.013	6.872
DISEASES ASSOCIATED WITH O-GLYCOSYLATION OF PROTEINS	2.72E-04	0.020	6.118
DISEASES OF GLYCOSYLATION	2.99E-04	0.020	4.134

Supplementary Table S9 Pathways linked to the changes observed in the RNA sequencing data of UOK276 cells treated with ODC$_B$. Significantly upregulated genes were entered into the Enrichr web-based analysis tool to generate this gene enrichment table from Reactome Pathways 2024. Only the first ten entries ranked by p-value are displayed.

TERM	P-VALUE	ADJUSTED P-VALUE	ODDS RATIO
AMPLIFICATION OF SIGNAL FROM UNATTACHED KINETOCHORES VIA A MAD2 INHIBITORY SIGNAL	7.56E-04	0.099	10.615
AMPLIFICATION OF SIGNAL FROM THE KINETOCHORES	7.56E-04	0.099	10.615
MITOTIC SPINDLE CHECKPOINT	0.001	0.106	8.921
EML4 AND NUDC IN MITOTIC SPINDLE FORMATION	0.002	0.106	8.520
RESOLUTION OF SISTER CHROMATID COHESION	0.002	0.106	7.818
HYALURONAN METABOLISM	0.002	0.106	31.213
RHO GTPASES ACTIVATE FORMINS	0.003	0.119	7.060
PLASMA LIPOPROTEIN ASSEMBLY, REMODELING, AND CLEARANCE	0.004	0.140	9.842
SEPARATION OF SISTER CHROMATIDS	0.007	0.216	5.499
MITOTIC PROMETAPHASE	0.012	0.277	4.774

Supplementary Table S10 Pathways linked to the changes observed in the RNA sequencing data of UOK276 cells treated with ODC$_B$. Significantly downregulated genes were entered into the Enrichr web-based analysis tool to generate this gene enrichment table from Reactome Pathways 2024. Only the first ten entries ranked by p-value are displayed.

TERM	P-VALUE	ADJUSTED P-VALUE	ODDS RATIO
EXTRACELLULAR MATRIX ORGANIZATION	7.74E-05	0.040	3.450
INTERFERON ALPHA BETA SIGNALING	2.11E-04	0.040	6.362
CYTOKINE SIGNALING IN IMMUNE SYSTEM	2.13E-04	0.040	2.304
CHEMOKINE RECEPTORS BIND CHEMOKINES	2.51E-04	0.040	7.575
DISEASES OF GLYCOSYLATION	4.76E-04	0.060	4.253
INTERLEUKIN-10 SIGNALING	7.16E-04	0.065	7.830
REGULATION OF IGF TRANSPORT AND UPTAKE BY INSULIN-LIKE GROWTH FACTOR BINDING PROTEINS (IGFBPS)	7.23E-04	0.065	4.455
DISEASES ASSOCIATED WITH O-GLYCOSYLATION OF PROTEINS	8.26E-04	0.065	5.939
PEPTIDE LIGAND-BINDING RECEPTORS	0.001	0.078	3.446
POST-TRANSLATIONAL PROTEIN PHOSPHORYLATION	0.001	0.087	4.511

Supplementary Table S11 Pathways linked to the changes observed in the RNA sequencing data of UOK276 cells treated with ODC$_C$. Significantly upregulated genes were entered into the Enrichr web-based analysis tool to generate this gene enrichment table from Reactome Pathways 2024. Only the first ten entries ranked by p-value are displayed.

TERM	P-VALUE	ADJUSTED P-VALUE	ODDS RATIO
FASL CD95L SIGNALING	0.023	0.396	54.084
G2 PHASE	0.023	0.396	54.084
MELANIN BIOSYNTHESIS	0.023	0.396	54.084
ALPHA-OXIDATION OF PHYTANATE	0.028	0.396	43.265
ASSEMBLY OF VIRAL COMPONENTS AT THE BUDDING SITE	0.028	0.396	43.265
FGFR3B LIGAND BINDING AND ACTIVATION	0.032	0.396	36.053
SYNTHESIS OF 12-EICOSATETRAENOIC ACID DERIVATIVES	0.032	0.396	36.053
FGFR1B LIGAND BINDING AND ACTIVATION	0.037	0.396	30.901
FUSION AND UNCOATING OF THE INFLUENZA VIRION	0.037	0.396	30.901
FUSION OF THE INFLUENZA VIRION TO THE HOST CELL ENDOSOME	0.037	0.396	30.901

Supplementary Table S12 Pathways linked to the changes observed in the RNA sequencing data of UOK276 cells treated with ODC$_C$. Significantly downregulated genes were entered into the Enrichr web-based analysis tool to generate this gene enrichment table from Reactome Pathways 2024. Only the first ten entries ranked by p-value are displayed.

TERM	P-VALUE	ADJUSTED P-VALUE	ODDS RATIO
EXTRACELLULAR MATRIX ORGANIZATION	1.71E-06	0.001	3.932
COLLAGEN FORMATION	2.95E-05	0.009	6.339
DEFECTIVE B3GALTL CAUSES PPS	4.89E-05	0.009	10.630
O-GLYCOSYLATION OF TSR DOMAIN-CONTAINING PROTEINS	5.69E-05	0.009	10.307
COLLAGEN BIOSYNTHESIS AND MODIFYING ENZYMES	1.73E-04	0.021	6.624
DISEASES ASSOCIATED WITH O-GLYCOSYLATION OF PROTEINS	2.49E-04	0.023	6.209
DISEASES OF GLYCOSYLATION	2.68E-04	0.023	4.195
INTEGRIN CELL SURFACE INTERACTIONS	7.50E-04	0.054	5.091
PEPTIDE LIGAND-BINDING RECEPTORS	7.99E-04	0.054	3.357
NEURONAL SYSTEM	0.001	0.058	2.480
